# Molecular Mechanisms for Changing Brain Connectivity in Mice and Humans

**DOI:** 10.3390/ijms242115840

**Published:** 2023-10-31

**Authors:** Pascale Voelker, Aldis P. Weible, Cristopher M. Niell, Mary K. Rothbart, Michael I. Posner

**Affiliations:** 1 Department of Psychology, University of Oregon, Eugene, OR 97403, USAmposner@uoregon.edu (M.I.P.); 2 Institute of Neuroscience, University of Oregon, Eugene, OR 97403, USA; aweible@uoregon.edu (A.P.W.); cniell@uoregon.edu (C.M.N.); 3 Department of Biology, University of Oregon, Eugene, OR 97403, USA

**Keywords:** anterior cingulate cortex, *Card6*, hippocampus, myelin, network training, *NF-κB*, nodal distance, state training, theta rhythm

## Abstract

The goal of this study was to examine commonalities in the molecular basis of learning in mice and humans. In previous work we have demonstrated that the anterior cingulate cortex (ACC) and hippocampus (HC) are involved in learning a two-choice visuospatial discrimination task. Here, we began by looking for candidate genes upregulated in mouse ACC and HC with learning. We then determined which of these were also upregulated in mouse blood. Finally, we used RT-PCR to compare candidate gene expression in mouse blood with that from humans following one of two forms of learning: a working memory task (network training) or meditation (a generalized training shown to change many networks). Two genes were upregulated in mice following learning: caspase recruitment domain-containing protein 6 (*Card6*) and inosine monophosphate dehydrogenase 2 (*Impdh2*). The *Impdh2* gene product catalyzes the first committed step of guanine nucleotide synthesis and is tightly linked to cell proliferation. The *Card6* gene product positively modulates signal transduction. In humans, *Card6* was significantly upregulated, and *Impdh2* trended toward upregulation with training. These genes have been shown to regulate pathways that influence nuclear factor kappa B (*NF-κB*), a factor previously found to be related to enhanced synaptic function and learning.

## 1. Introduction

Learning has been shown to modify brain circuitry through experience-dependent plasticity [1]. Our understanding of learning circuits in humans has increased dramatically in the last several decades [2]. However, the temporal and spatial resolution of the techniques available limit the granularity of that understanding. In contrast, animal studies have enabled a more direct manipulation of these same circuits in order to observe molecular and genetic function [3,4]. By examining commonalities and differences in genetic regulation following learning in each system, we hope to further understand how studies of mice may improve our understanding of the molecular basis of human learning.

Brain networks, such as that of attention, are composed of nodes distributed among cortical and subcortical regions [5]. Connectivity between these nodes can be modified through learning, as, for example, with changes in the periaxonal space and the distance between nodes [6], as well as through myelination [7]. In humans, the two broad categories of brain training that induce changes in connectivity are network training and state training [8]. Network training involves conventional practice of one or more cognitive tasks, such as, for example, acquisition of a working memory task [9]. A second method of altering the brain is one in which the brain state might be changed, as with the practice of meditation [10]. 

There is extensive research going back decades linking the hippocampus (HC) with memory, and recent reviews discuss the role of the anterior cingulate cortex (ACC) in learning and memory [11,12]. In order to support skill learning, attention networks need to connect to memory networks. One pathway between attention networks and memory networks connects the ACC with the HC through the nucleus reuniens of the thalamus [11]. In a mouse study, this route has been shown to be important in the generalization of fear conditioning [13]. Whether this pathway is involved in the generalization of skills other than fear conditioning is unknown. However, human studies using MRI have shown that the ACC and HC are both activated in a variety of tasks [14,15]. A second pathway between the attention and memory networks includes the entorhinal cortex (EC) and parietal lobe [16]. There may be overlap between the two pathways because the nucleus reuniens is also connected to the HC through the entorhinal cortex [11]. 

In a previous study, we identified the involvement of the mouse ACC and HC during learning of a two-choice visuospatial discrimination task [17], which is an example of network training [18]. In this task, a trained group learns to associate the position of a visual cue with a rewarded motor response. In studies of human learning, we have compared methods of training to specific changes in brain function [18]. State training, in this case meditation, has been associated with changes in connectivity [19] and cognitive function [20], which may reflect the changes seen from network training, such as memory [21,22].

Our previous work indicated similarities in the brain pathways linking the attention and memory networks in mice and humans [11]. These links include connections from the ACC through the thalamus to the HC and from the posterior parietal lobe through the entorhinal cortex to the HC [10]. The HC has been regarded as the primary brain region facilitating episodic or declarative memory, and recent studies have clarified the essential role of the ACC in the recall of remote memories [12,23,24,25]. During an inhibitory avoidance memory paradigm, increases in immediate early gene expression were detected not only in the amygdala and HC, but also in the medial prefrontal cortex (mPFC) and ACC [26]. Similarly, gene expression in these four brain regions was required for the consolidation of social recognition memory [27]. Although the transcriptomic profiles of the HC and ACC are significantly different from one another, it appears that learning a task induces conserved programs of gene expression [28]. Commonalities in induced gene expression between these tissues would represent a conserved system, which may be detectable in blood [29]. In fact, blood biomarkers have been used in many studies to examine brain health and function [23,24,25,26,27,28,29,30,31,32,33], and while protein biomarkers are most common, RNA biomarkers have also been identified [34,35,36,37,38,39].

Based on these studies, we hypothesized that some genes upregulated in the mouse brain and blood may also be upregulated in human blood following learning. Our goal was to determine whether the key brain regions required for learning demonstrate similar changes in gene expression which can be detected in blood. To do so, our first objective was to identify candidate genes through gene expression profiling, using RNA isolated from the ACC and HC in trained and control mice to probe transcriptional microarrays. Our second objective was to determine whether any of the candidate genes most strongly upregulated with training in both the ACC and HC were also upregulated in the blood after training. Finally, we wanted to determine whether these candidates also varied with training in humans.

In addition to predicting the existence of training-specific changes in gene expression in the blood, we expected that different kinds of training may induce slightly different gene expression profiles. Differences may be identified by assaying blood from humans undergoing either network training (a working memory task) or state training (mindfulness meditation) for changes in gene regulation of these candidates. We proposed that working memory training, which has been associated with activation of the HC, may alter the expression patterns in blood of the genes involved in HC function. There is some precedence for this, as exemplified by the positive correlation between brain-derived neurotrophic factor (BDNF) levels in the blood and brain across species [40]. BDNF is critical to adult hippocampal neurogenesis during learning [41]. Similarly, meditation training, which has been associated with ACC activation, could correlate with the changes in gene expression in the blood associated with ACC function. We hope that our results, from looking at candidate gene expressions, will help reveal the pathways and mechanisms influential in different types of human learning.

## 2. Results

### 2.1. Training

**Figure 1:**
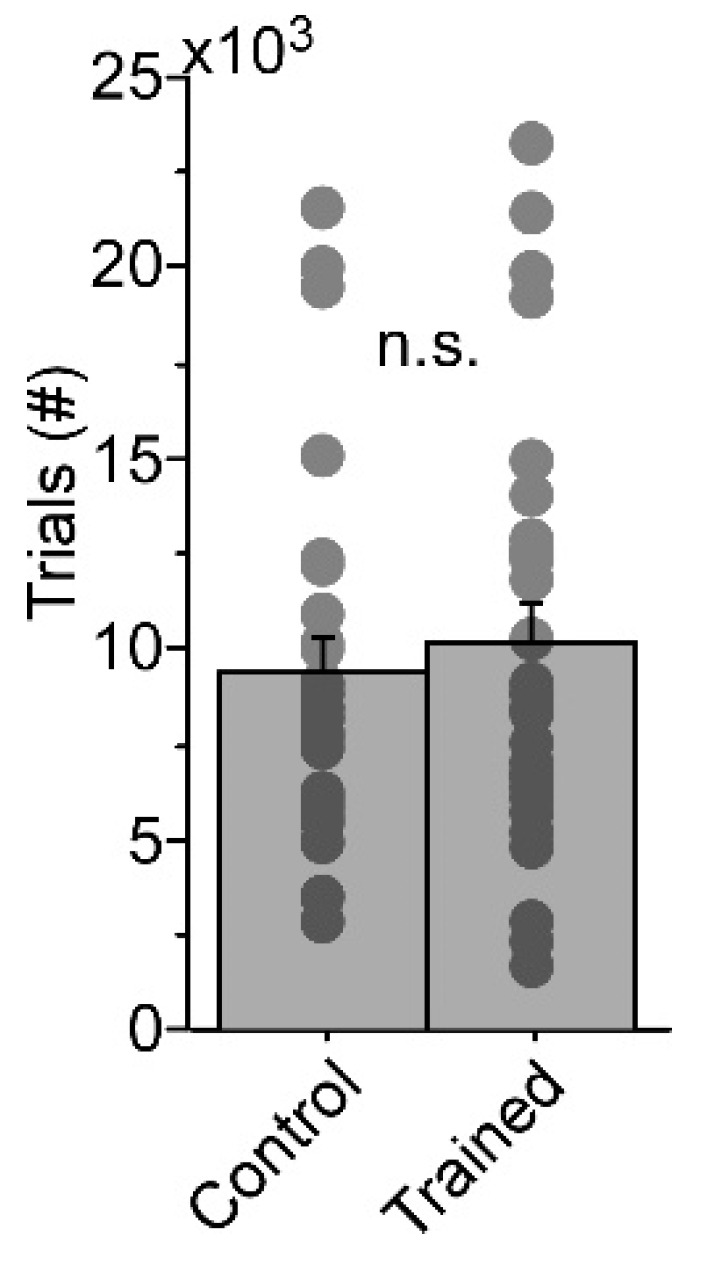
Total number of trials for trained and control mice. Individual trials-to-criterion are displayed as dots. Mean ± SE; n.s.: not significant.

Mice were trained in a two-choice visuospatial discrimination task [17]. Water-restricted mice were trained in pairs, with one mouse required to learn an association between a cue position and the direction run in response to receive a water reward (“trained”), while the other mouse was randomly given water over the course of the session irrespective of cue position or motor output (“control”). After the trained mouse reached the behavioral criterion of >85% correct responses during a block of 50 consecutive trials, both mice were sacrificed and their blood and brain tissue were harvested for subsequent genetic analysis (see Section 4). Each group included 30 mice, and the pairs were same-sex littermates (trained: 16F, 14M; control: 16F, 14M). The average days-of-training to criterion was 27 ± 2.6. While paired mice received the same number of days of training, the trials/session varied between groups as trained mice typically required more trials to achieve satiety during the initial stages of learning. However, this difference in total trials was not significant (Figure 1).

### 2.2. Gene Expression in the Mouse Brain

**Figure 2:**
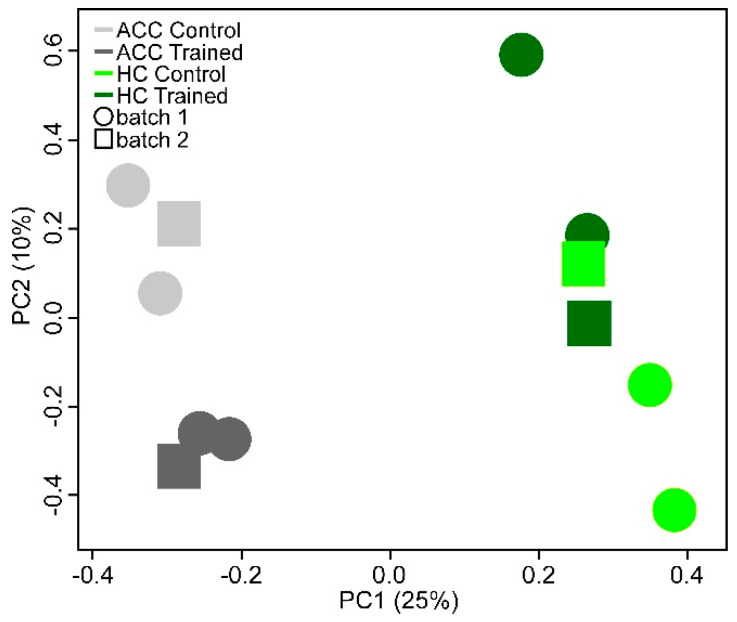
Graph of principal component analysis of the mouse transcriptional microarrays showing that variation is accounted for by brain region (PC1) and training status (PC2).

The transcriptional expression profile of the ACC and HC was determined from brain tissue harvested from trained and control mice. A principal component analysis (PCA) was used to compare the similarities and differences in gene expression with learning (trained vs. control) (Figure 2). The first principal component (x-axis) shows that expression was more similar within a brain region compared to between regions, which explains 25% of the variance. The second principal component (y-axis) shows that expression was grouped by training status, which explains 10% of the variance. The separation along the y-axis is stronger for the ACC samples than the HC samples. Because of the large number of genes assayed, the threshold for significance following a correction for multiple comparisons (q) exceeded even our most individually significant (p) observations. This is in line with observations from other studies [42]. For our purposes, upregulation with training was determined by comparing rank order scores > 2, which represented approximately the top 5% of genes sorted for upregulation. We identified 25 candidate genes upregulated in both the ACC and HC (Table S1 of the Supplementary Materials).

### 2.3. Gene Expression in Mouse Blood

Next, we assayed blood from the mice to identify the genes upregulated with training. The rank scores related to the strength of expression in blood were then compared to that of the brain tissue. While 25 genes in both HC and ACC had rank order values > 2, only three of these were also >2 in blood (Table 1). These three genes were selected for further analysis.

We combined two independent mouse experiments (two arrays/condition and one array/condition) in a new analysis so that we had biological replications in triplicate for each condition, which could improve the detection of true positives and be used to weigh the likelihood of candidate genes. Table 2 summarizes the microarray intensity data from both experiments for the three genes with >2 rankings in both the brain and blood. This comparison supports upregulation due to learning for Caspase Recruitment Domain-containing protein 6 (*Card6*) and Deoxyribonuclease-1-like 2 (*Dnase1l2*). However, the data do not hold true for Inosine Monophosphate Dehydrogenase 2 (*Impdh2*) when compared to the original rank order or intensity data. This reversal may be due to noise in the microarray detection (since only one microarray was assayed in the second experiment), or it could be a genuine divergence in expression among the pooled individuals in this group. Also, at this stage we did not know whether all pooled individuals showed similar expression within the pool or whether a few outliers showed greater gene expression changes. From the literature we determined that *Card6*, *Impdh2*, and perhaps *Dnase1l2*, may act to regulate Nuclear Factor Kappa B (*NF-κB*) [43,44,45], so we also present the expression of a pair of genes important to *NF-κB* expression [Inhibitor Of Nuclear Factor Kappa B Kinase Subunit Beta (*Ikbkb*) and Nuclear Factor Kappa B2 (*Nfkb2*)]. We also present the expression of interferon regulatory factor 8 (*Irf8*), which has been shown to be upregulated in blood via meditation in another study [46]. Tyrosine 3-Monooxygenase/Tryptophan 5-Monooxygenase Activation Protein Zeta (*Ywhaz*) [47,48,49], our reference gene described below, showed consistent expression across conditions.

### 2.4. Mouse RT-PCR

**Figure 3:**
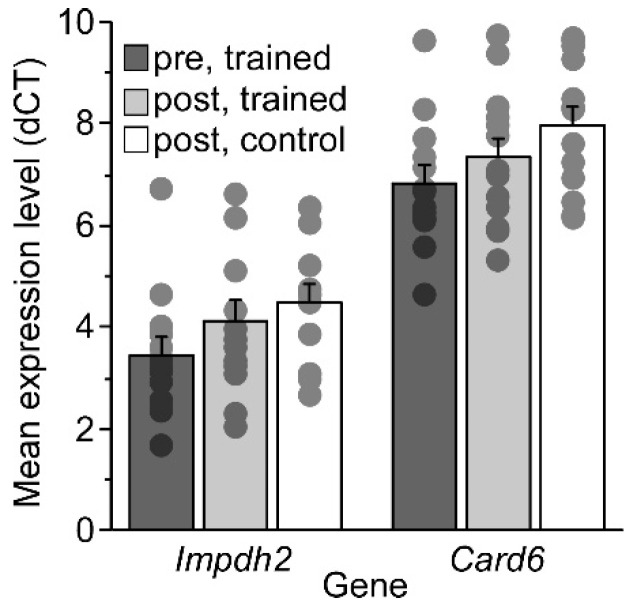
RT-PCR results showing average dCt values of mouse gene expression in blood as a function of their group (lower threshold values show stronger expression). Individual values are displayed as dots.

Next, the upregulation of two of the genes common to the mouse brain and blood were corroborated using real-time PCR (RT-PCR) amplification. Individual RNA samples were amplified using quantitative amplification relative to an internal reference gene, *Ywhaz*. *Ywhaz* is considered to be a housekeeping gene, performing functions in vital cellular processes such as metabolism, apoptosis, and the cell cycle, and is highly conserved between species. *Ywhaz* demonstrated moderate expression levels in RT-PCR and showed little variability in expression on our microarrays (across all arrays for mean log2 intensity ACC: 6.4+/−0.04, HC: 6.3+/−0.1 blood: 4.0+/−0.1). We found that *Card6* and *Impdh2* appeared to be upregulated in most individual samples relative to the control (of 13 trained/control pairs, *Card6* was upregulated in 9; *Impdh2*, upregulated in 8). This is consistent with the microarray data from the first experiment, which used pools of these individual samples and had high rank scores for these genes. In Figure 3, mean expression is shown as a function of the training condition. For both genes, the “post, trained” group showed higher expression levels (shown in Figure 3 as a lower delta cycle threshold (dCt) value) following training compared with the age-matched controls. The highest mean expression level was prior to training, presumably showing the strong age effect on these genes. An analysis of variance showed significant differences between groups (*Card6*: F(2,24) = 7.20 *p* = 0.0036; *Impdh2*: F(2,24) = 3.86 *p* = 0.035), and pairwise comparisons showed that the “post, control” group differed from the “pre, trained” group (*Card6*: t(12) = 4.30 *p* = 0.001; *Impdh2*: t(12) = 3.07 *p* = 0.0097) whereas the “post, trained” group did not (*Card6*: t(12) = 1.62 *p* = 0.13; *Impdh2*: t(12) = 1.62 *p* = 0.13). This indicates that there is a significant decline in *Card6* and *Impdh2* transcript levels over time that is attenuated with training. Unfortunately, *Dnase1l2* was not tested as a result of technical issues beyond our control. 

### 2.5. Genetic Expression in Human Blood

We then examined the three candidate genes upregulated in the mouse brain and blood in human blood. Because the human training lasted only two weeks and the participants were 18+ years old, we assumed there would be little to no effect of age on gene expression, and thus compared within-subject levels of expression. Each row is a different participant in Table 3, and it is possible to use a count of positive (downregulated) vs. negative (upregulated) numbers to determine significance, on the assumption that up- and downregulation would be equal unless influenced by learning.

**Figure 4:**
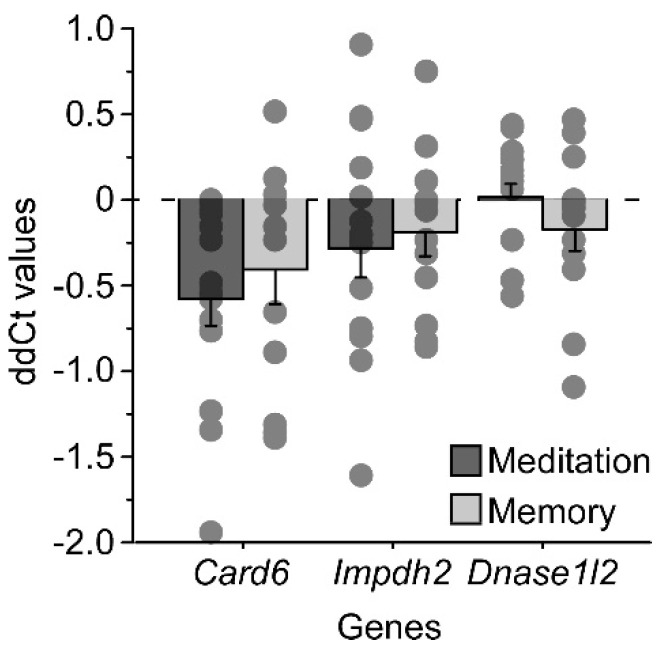
RT-PCR results showing average ddCt values of human gene expression in blood as a function of gene and training method (lower threshold values show larger increases in expression). Individual values are displayed as dots.

Humans underwent either network training (working memory) or state training (meditation). As shown in Table 3 and Figure 4, *Card6* was significantly upregulated following meditation (F(1,13) = 13.9 *p* = 0.0025) and showed a similar trend following working memory (F(1,11) = 3.98 *p* = 0.071). *Impdh2* tended toward upregulation (meditation: F(1,13) = 2.33 *p* = 0.15; memory: F(1,11) = 1.88 *p* = 0.199) in both tasks, while *Dnase1l2* showed no upregulation following memory training (F(1,11) = 1.49 *p* = 0.25) or meditation (F(1,13) = 0.01 *p* = 0.92) (Figure 4).

We also investigated two genes facilitating the upregulation of *NF-κB* in our human blood samples, *NF-κB2* and *Ikbkb*, in a subset of our human samples. Looking back onthe microarray data from mice, we see that these genes are only upregulated in blood (blood: Table 2, brain intensities not shown). In humans, *NF-κB2* and *Ikbkb* showed a pattern of upregulation in the working memory group and downregulation in the meditation group (Table 4). One gene in the interferon pathway which has previously been shown to be upregulated with meditation training [46] is *Irf8*, which regulates transcription in response to interferon signaling. In our mice, this gene was upregulated in blood and, to a lesser degree, in both the ACC and HC (blood: Table 2, brain intensities not shown). In our subset of human samples, *NF-κB2* trended toward downregulation in the meditation group (F(1,16) = 3.76 *p* = 0.07). In general, transcription of the genes trended towards upregulation in our memory group (*n* = 7) and downregulation in our meditation group (n = 9), though this was not significant (Table 4).

Overall, we have found one gene, *Card6*, that was upregulated in the brain of mice and the blood of both humans and mice. Another gene, *Impdh2*, trended towards upregulation in human blood and in the mouse brain, and was seen via RT-PCR to also trend towards upregulation in mouse blood, but it did not repeat in the third microarray replicate. Both of these genes have been found previously to influence the expression of the *NF-κB* factor, which has been shown to be critical to learning.

*Dnase1l2* showed consistent evidence for a modest upregulation in both mouse brain and blood with training. However, only a trend was observed in humans, and only with network training. We are unaware of any previous reports of a relationship between *Dnase1l2* upregulation and learning in mice or humans.

### 2.6. Pathways

We next looked at coordinated changes in the expression of genes grouped by their function in pathways using Gene Set Enrichment Analysis (GSEA) [42]. As described below, these findings lead us to associate the involvement of collagen and *NF-κB* with learning.

We performed a GSEA to test whether changes in gene regulation were associated with specific pathways. A GSEA can detect more subtle changes in gene expression, as this analysis is built on the connection between an annotated gene function and its contribution to biochemical pathways. The GSEA output can show high redundancy because it draws on annotation from several databases and specific gene products can have multiple functions. In the brain, we did not see significant changes in pathway expression related to training alone, but there was a region x training effect, where five pathways had a significant (*q* < 0.05) change in regulation with training, and were upregulated in the HC but downregulated in the ACC. All five of these genes relate to collagen metabolism (Table S2 of the Supplementary Materials). In contrast, the blood GSEA, comparing trained to control mice after the training period, had 814 pathways enriched at *q* < 0.05 (810 upregulated). Importantly, no pathways were significantly enriched before the training period between these two groups. As a matter of interest, a pre–post comparison of trained mice demonstrated 163 enriched pathways (100 upregulated) at *q* < 0.05, whereas that of untrained mice showed 870 enriched pathways (134 upregulated). Thus, the bulk change in gene regulation was downregulation over the training period, but training exerted a 10-fold attenuation of this effect (trained: 63 downregulated vs. control: 736 downregulated).

**Figure 5:**
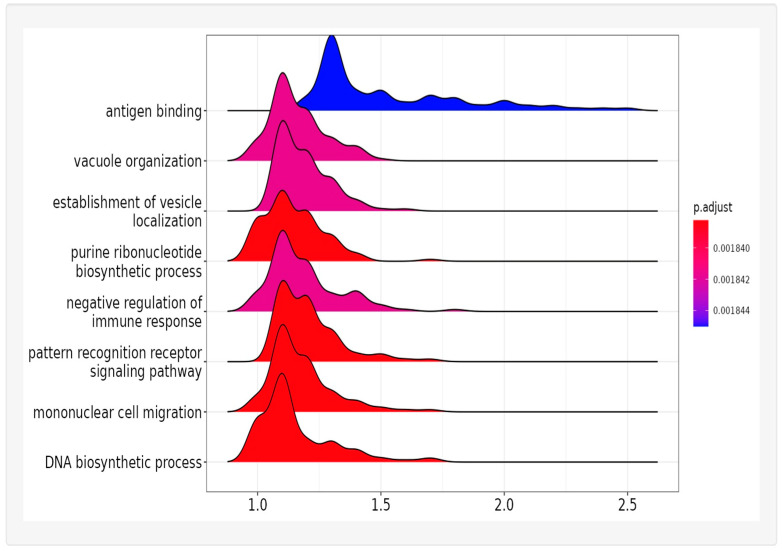
Top eight differences in gene expression when comparing trained to control mice using ClusterProfShinyGSEA [49]. The color indicates the *p*-value significance, the x-axis designates up- (positive) or down- (negative) regulation.

Since the GSEA produces a list of highly redundant pathways, a further analysis was performed to characterize the type of significantly upregulated pathways observed. Using ClusterProfShinyGSEA [50], our list of mouse genes ranked by upregulation due to training is represented by the eight most significantly upregulated systems: antigen binding, vacuole organization, vesicle localization, purine ribonucleotide biosynthesis, negative regulation of immune response, pattern recognition receptor signaling, mononuclear cell migration, and DNA biosynthesis (Figure 5). These systems could be summarized by the activation of immunity and cell proliferation. Some of these systems directly implicate changes in gene expression/cell activity/cell shape, which might be expected during the process of neural restructuring. A key player in these processes is *NF-κB*, which coordinates the immune and metabolic systems [51]. The greatest change in pathway regulation was the positive regulation of cell activation and included those pathways specific to *NF-κB* regulation. Of the 810 pathways enriched due to training (Table S3), many of these involve *NF-κB*.

## 3. Discussion

Our goal in this study was to identify the learning-related changes in gene expression common to mice and humans. To accomplish this, we assayed for genes upregulated with training in the mouse brain (ACC, HC) and blood samples, and identified three candidates: *Card6*, *Impdh2* and *Dnase1l2*. We also assayed the three selected genes before and after learning in the blood of humans. While blood may not represent the full array of changes found in the brain, our mouse work reinforces the assertion that blood is a workable proxy for changes in the brain. Indeed, we found evidence of the upregulation of genes in human blood that were also found to be upregulated in the ACC and HC of mice.

It is possible to compare the size of the upregulation in blood by investigating the fold change in both species, although different techniques were used and we are comparing means of pooled samples (microarray) and individual samples (RT-PCR). In mouse blood, the *Card6* gene was upregulated 1.60× (microarray) and 1.57× (RT-PCR) by learning, representing a comparable range. In humans, working memory training upregulated *Card6* 1.32× (RT-PCR) and meditation 1.50× (RT-PCR). For *Dnase1l2*, the change in mice was 1.20× (microarray), while humans in the working memory group showed a change of 1.12× (RT-PCR) (meditation was unchanged at 0.99×; RT-PCR). For *Impdh2*, overall mouse blood was downregulated by −1.20 (microarray) and upregulated 1.25× (RT-PCR) while the change in human blood was 1.14× for memory training (RT-PCR) and 1.21× for meditation (RT-PCR). As mentioned above, the inconsistency between the microarray data and RT-PCR for *Impdh2* may be due to technical factors, since two of the three arrays averaged 1.9× upregulation. Alternatively, *Impdh2* may show larger variances in expression. In humans, meditation showed a significant effect for *Card6* and was marginally upregulated with memory training. *Impdh2* trended in the same direction, but not to significance.

### 3.1. Mice

Pre-training blood was collected from mice 1 week prior to the first session (at the time of surgery). Mice were then trained for an average of 27 days to criterion performance. Since the mice averaged 75 days of age at the time of surgery, this would represent a greater part of their life cycle compared to the human subjects. Differences in the regulation of genes due to aging in mice [52] could obscure the changes due to learning. This may be evidenced in Figure 2, in which pre-trained mice showed the greatest expression (lowest threshold) for both genes. To address this, trained mice were paired with age- and sex-matched controls. Since aging was the same in both groups, differences due to learning would be apparent by comparing the trained and control groups. We found a significant effect of learning on *Card6* and a modest effect of learning on *Impdh2*.

### 3.2. Humans

Since the human participants were all over 18 years old and the time between blood draws was two weeks, we assume that gene regulation did not fluctuate relative to aging. To make sure that differences were due to learning and not to other factors we examined the difference scores in ddCT values (before learning subtracted from those after learning). We used two human tasks which may be viewed as two ways to elicit training-related changes in functional connectivity. One was a working memory task, which is a typical method for changing the efficiency of memory networks [53]. The other was a mindfulness meditation practice that has been shown behaviorally [20], as well as by imaging [54], to alter many quite separate networks, such as those underpinning attention and memory processes, and has been shown via diffusion tensor imaging to change the white matter surrounding the ACC [19].

We found evidence of upregulation in human blood of two of the three genes found to be upregulated in the ACC and HC in our trained mice. These were *Card6* and *Impdh2*. The third gene, *Dnase1l2*, showed small upregulation due to working memory training but not meditation. The *Card6* gene was found to be upregulated due to meditation training in all 14 participants. This effect was clearly significant, while upregulation of *Impdh2* was less robust. In working memory training both *Card6* and *Impdh2* were upregulated in 8 of 12 participants (probability 0.12).

We have clear evidence for upregulation of *Card6*, and likely also *Impdh2*, in human learning. The amount of upregulation in human blood was comparable between working memory and meditation training. This led to a rejection of the hypothesis that working memory training would involve the upregulation of a different set of individual genes than meditation; however, judgement based on three genes is hardly conclusive since learning is expected to influence multiple pathways and involve many genes, only some of which might be detectable in the blood.

We tested an additional three genes due to their potential functional relationship to *Card6* and *Impdh2* (*Ikbkb, NF-κB2*) and a previous study involving meditation (*Irf8*; [46]). While working memory training showed some upregulation of these genes and meditation showed some downregulation, there were not sufficient data for a meaningful significance test, and further investigation is warranted. This could represent a dissociation in the functional pathways between the two training methods.

### 3.3. Common Pathway

Collagen, as part of the extracellular matrix, plays an important role in the morphogenesis of neural tissue [55]. In the brain, collagen is involved in functions such as synaptogenesis, axonal guidance, and cell adhesion [56,57]. It also plays a major role in neural maturation [58]. Collagen peptides were shown to enhance neurogenesis in the HC and reduce anxiety-like behavior in mice [59]. Research demonstrated that implanting collagen can be used to remediate traumatic brain injury by supporting neural regeneration and reducing cognitive loss [60]. It is interesting that, in mice, the HC contrasts with the ACC in collagen expression. We hypothesize that collagen may play a larger role in HC neural restructuring with learning, the restructuring may be more extensive, and/or the timeframe of restructuring could be different than in the ACC.

Our observation that mouse blood, rather than the brain, shows greater changes in gene expression with training is important for the development of biomarkers which could be used to monitor training. We concentrated on biomarkers which would be common between humans and mice and identified one prime candidate: *Card6*. We think that this upregulation with learning is indicative of neuroplastic activity in the brain. In general, we expect that only rate-limiting steps in pathways stimulated by learning would show dynamic expression, but it is possible that with repeated training more elements would be affected.

The *Card6* gene contains a caspase-recruitment domain (CARD) facilitating protein–protein interactions. The encoded protein is microtubule-associated and positively modulates signal transduction, which leads to activation of *NF-κB* [61]. Recent work demonstrated that the over-expression of *Card6* suppressed rheumatoid inflammation and *NF-κB* activity [62]. *Card6* was an important protective factor in spinal cord injury progression, limiting the inflammatory response in mice by suppressing *NF-κB*-mediated enhancement of pro-inflammatory cytokines [63].

*Impdh2*, also identified in our analysis, is associated with learning to a lesser extent. The *Impdh2* gene encodes a rate-limiting enzyme in de novo guanine nucleotide synthesis, maintaining the pools available for RNA and DNA synthesis [64]. Important to cell growth, it has been upregulated in some neoplasms. A dominant mutation of *Impdh2* has recently been associated with dystonia linked with dopamine biosynthesis [65]. *Impdh2* has been shown to have high affinity to *Park2* [66], mutations in which are a major cause of early onset Parkinson’s disease, a progressive neurodegenerative disorder characterized by a selective loss of dopaminergic neurons. *Impdh2* was shown to mediate SARS-Co-V2 activation of *NF-κB* [67] and is a target for the immunosuppression of neuroinflammatory disease [68]. In this group it was observed that *NF-κB* activity was suppressed when *Impdh2* expression was knocked-down. Conversely, *Impdh2* is likely a transcriptional target of *NF-κB* [69,70], thus demonstrating an intimate co-regulation with *NF-κB*. Thus, our candidate genes point to the involvement of *NF-κB* regulation. The literature has implicated *NF-κB* in learning [71]. It is possible that *Card6* and *Impdh2* represent a rate-limiting or enhancing function in *NF-κB* regulation.

*NF-κB* functions in cell activation, and was shown to be involved in both immunological and learning mechanisms [71]. *NF-κB* is highly conserved and has been observed, in fruit flies, mice, and humans, to induce LTP in the hippocampus. More than 20 years of research on *NF-κB* in the nervous system has yielded evidence of the involvement of *NF-κB* target genes in synaptic enhancement [72]. Our blood GSEA detected the upregulation of immunity-related pathways which also involve the activity of *NF-κB*. We hypothesize that these *NF-κB*-related systems actually play a role in biological changes, facilitating learning in this case, rather than the annotated functions in innate and adaptive immunity. There is a growing argument that normal homeostatic and immune-related gene expression is intricately linked [73], a feature not generally reflected in current annotations. If so, our blood GSEA specifically points to a learning-related restructuring of neural systems.

A different method of relating mice and humans on similarities at the molecular level examines rodent models of human degenerative diseases such as Parkinson’s [74] and Alzheimer’s disorders [75]. Although differing in method and aims from our approach, a related study has uncovered cross talk between the *NF-κB* and *Nrf2* pathways in the regulation of inflammation and oxidative stress [76]. This relationship needs further analysis to determine whether it may help in understanding the molecular mechanisms of learning and memory.

Our mouse data, comparing trained and control mice of the same age, show strong upregulation of the *NF-κB*-related factors. We also found strong effects of aging on this pathway when comparing mouse blood pre- and post-training. As has been reported previously, mice generally show changes in gene expression with age [52]. We found that training decreased downregulation, indicating a learning-related gene regulation influencing *NF-κB* activity.

The GSEA identified several pathways related to immunity. Antigen binding is an example of adaptive immunity, and monocytes are part of the innate immune system. The negative regulation of an immune response can regulate both innate and adaptive immunity [77]. Pattern recognition receptors recognize specific molecular structures and can facilitate an immune response. The adaptive and innate immune systems have been shown to interact with brain development and function and can play a role in brain dysfunction [78]. Deficits in the brain–immune interaction can result in impairments in synaptic plasticity [79]. The immune system is important to normal brain functioning, for example, microglial cytokine activity modulates synaptic plasticity [80]. Immune-deficient mice have been shown to have cognitive impairments [81]. Likewise, in humans, the adaptive immune system plays an important role in hippocampal neurogenesis throughout life [82].

Also prevalent in the GSEA, vacuole organization plays an important role in sequestering harmful metabolic products and in exocytosis, the delivery of products to regions external to the cell [83]. Vesicles transport molecules during the process of exocytosis. Vesicle localization directs the movement of molecules, as would happen during organelle formation or synaptogenesis [84]. Synaptic vesicles facilitate neurotransmitter release. We do not know the exact purpose of vacuole organization and vesicle localization, but they are consistent with immune function and neurogenic activity.

Finally, the GSEA also detected DNA biosynthetic processes, indicative of cell division, while ribonucleotide biosynthetic processes indicate the production of mRNA, which facilitates gene expression. Together, these are indicators of cell proliferation. Cell proliferation and migration can be a sign of immune function, and can also support adult neurogenesis [85]. In sum, this analysis has indicated an increase in immunity functions and cell proliferation, which may act to facilitate the neural plasticity which would need to occur during the process of learning.

One caveat to this analysis is that we are looking for changes in gene expression in a medium remote from brain activity: blood. Activity in the brain is not wholly isolated from the circulatory system. One better characterized example is neuro-immune bi-directional interactions. Immune reactions have been shown to impact brain function and behavior [86] and psychological properties have been demonstrated to modulate immunity [87]. A whole genome analysis comparing transcription in human blood and cerebellum determined that 22% of transcripts had highly correlated (r = 0.98) expression levels [88]. Another group characterized the correlation of gene networks rather than single genes in a comparison between blood and the cortex, caudate nucleus, and cerebellum [29]. They found a greater correlation of gene expression between blood and the cortex, and significant preservation of the systems involved in immunity and gene expression. So while some neurogenic functions may be isolated within the brain, other relevant signals and responses have been identified in the blood.

## 4. Materials and Methods

### 4.1. Mice

All procedures were conducted in accordance with the ethical guidelines of the National Institutes of Health and were approved by the Institutional Animal Care and Use Committee at the University of Oregon. Animals were maintained on a reverse 12/12 h light/dark cycle. Training and experiments were performed during the dark phase of the cycle. All mice (28 male, 32 female) were 8–12 weeks of age at the time of surgery, and were bred from the C57Bl6/J background strain.

### 4.2. Surgery

We administered atropine (0.03 mg/kg) pre-operatively to reduce inflammation and respiratory irregularities. Surgical anesthesia was induced and maintained with isoflurane (1.25–2.0%). The pre-training blood sample was collected at the time of surgery. A titanium cross-bar was also fixed to the skull to enable restraint of the mouse during behavior training. The cross-bar was cemented in place with Grip Cement. Carpofen (10 mg/kg) was administered post-operatively to minimize discomfort. Mice were housed individually after the surgery and allowed 7 d of postoperative recovery.

### 4.3. Training and Sample Harvesting

Mice were trained as described previously [17]. Briefly, water-deprived mice were fixed in position via the titanium cross-bar on an air-cushioned Styrofoam ball, facing a vertically oriented computer monitor on which visual cues were presented at the top or bottom of the screen. A spout was placed immediately in front of the mouse for delivery of a water reward. Trained mice learned to run left for a cue presented at the top of the screen and right for a cue at the bottom for a water reward. Control mice were presented with the same visual stimuli, but water was presented at random intervals. Mice were weighed for three days prior to training to establish a baseline weight, then weighed daily before and after training to determine the amount of water consumed. If a mouse dropped to 80% of baseline, it was given a supplement of pulverized breeder chow mixed with 1ml water after the day’s training. Pairs of mice were given daily sessions until the trained mouse achieved a behavioral criterion of >85% correct responses during a block of 50 consecutive trials. The pre-training blood sample was taken during surgery. A small nick was made using a scalpel in the bone overlying the fronto-nasal sinus immediately posterior to the olfactory bulb. Typically, 100–150 μL of blood was collected with a pipetter and transferred immediately to an aliquot containing 600 μL RNAlater on ice. Post-training blood and tissue were harvested within 24 h of criterion performance. Both trained and control mice were sacrificed using an overdose of Isofluorane. Mice were decapitated, and blood (100–200 μL) was taken upon decapitation, immediately prior to harvesting of tissue from the ACC and HC, and was pipetted from the trunk into an aliquot containing RNAlater on ice. The brain was then removed and immersed immediately into a bath of chilled RNAlater. Using a pair of opthalmic scalpels, the brain was hemisected and the ACC and HC dissected bilaterally and placed in separate aliquots containing 200 μL RNAlater. To summarize, we had blood samples collected from the trained and control mice before training, and we had all samples (blood, ACC, HC) from the experimental and control mice after training.

### 4.4. RNA Preparation/Microarray

The brain tissue was dissected, trimmed, and stored in a bath of RNAlater (Invitrogen, Carlesbad, CA, USA). Each sample was first manually homogenized in liquid nitrogen, followed by finer homogenization using a QIAshredder column (Qiagen, Germantown, MD, USA) before RNA isolation. RNA was isolated from each sample following the kit instructions (RNeasy, Qiagen, Germantown, MD, USA). Blood was collected and immediately stored in RNAlater. RNA from each sample was isolated using the Mouse RiboPure-Blood RNA Isolation Kit (Invitrogen, Carlesbad, CA, USA). Every RNA sample was quantified and quality-scored using an Advanced Analytical Fragment Analyzer through the Genomics and Cell Characterization Core Facility, UO. The best quality samples (RQN > 7) were pooled together in groups in order to look at group differences in gene expression related to learning in each brain region and in blood. Each condition had two unique pools containing equal quantities of each RNA sample, thus were biological replicates. The HC and blood pools each contained RNA from 7 mice, the ACC each had RNA from 5 mice due to the difficulty isolating high quality RNA from this small tissue sample. Pooled samples originating from blood were further processed by removing excess globin transcripts which could interfere with sensitive transcript detection (GlobinClear, Ambion, Naugatuck, CT, USA). These pooled samples were also analyzed to insure sample quality (RIN > 7), then probed onto mouse gene expression microarrays (mouse 2.0ST, Affymetrix, Santa Clara, CA, USA) in order to profile the differences. This work was carried out by the Boston University Medical Campus Microarray and Sequencing Resource, which also provided computational analysis of the microarray data. After a period of time, we repeated this whole experiment with one pool/microarray for each condition, the same facility analyzed the new data along with the previous experiment so that we had biological triplicates for each condition.

### 4.5. Microarray Analysis

Mouse Gene 2.0 ST CEL files were normalized to produce gene-level expression values with the Robust Multiarray Average (RMA) [89] using the affy R package (version 1.62.0) and Entrez Gene-specific R packages (version 24.0.0) from the Molecular and Behavioral Neuroscience Institute (Brainarray) at the University of Michigan [90]. Gene biotypes were obtained using the biomaRt R package (version 2.39.4). Gene Ontology (GO) terms and Kyoto Encyclopedia of Genes and Genomes (KEGG) pathways were obtained using the GO.db and KEGG.db packages (versions 3.8.2 and 3.2.3, respectively). Array quality was assessed by computing the Relative Log Expression (RLE) and Normalized Unscaled Standard Error (NUSE) using the affyPLM R package (version 1.59.0). Principal component analysis (PCA) was performed using the prcomp R function with expression values that had been normalized across all samples to a mean of zero and a standard deviation of one. Differential expression was assessed using the moderated (empirical Bayesian) *t*-test implemented in the limma R package (version 3.39.19) (i.e., creating simple linear models with lmFit, followed by empirical Bayesian adjustment with eBayes). Correction for multiple hypothesis testing was accomplished using the Benjamini–Hochberg false discovery rate (FDR). Human homologs of mouse genes were identified using HomoloGene (version 68). All microarray analyses were performed using the R environment for statistical computing (version 3.6.0). Candidate genes were selected on the basis of passing a threshold, because individual genes did not meet significance for multiple testing. Since false positives can be mistaken for genuine candidates, our strategy was to inform our selection of candidates with relevant data from gene expression in the brain.

### 4.6. Gene Set Enrichment Analysis (GSEA)

GSEA (version 2.2.1) [42] was used to identify biological terms, pathways, and processes that are coordinately up- or downregulated within each pairwise comparison. The Entrez Gene identifiers of the human homologs of the genes interrogated using the array were ranked according to the t-statistic computed for each effect in the two-factor model, as well as for each pairwise comparison. Any mouse genes with multiple human homologs (or vice versa) were removed prior to ranking, so that the ranked list represents only those human genes that match exactly one mouse gene. Each ranked list was then used to perform pre-ranked GSEA analyses (default parameters with random seed 1234) using the Entrez Gene versions of the Hallmark, Biocarta, KEGG, Reactome, Gene Ontology (GO), and transcription factor and microRNA motif gene sets obtained from the Molecular Signatures Database (MSigDB), version 7.5.1.

### 4.7. Real-Time PCR (Mouse)

Individual RNA samples were reverse transcribed using the Maxima First Strand cDNA Synthesis kit (Thermo Fisher, Waltham, MA, USA). This product was subsequently amplified using relative quantification to an internal reference gene, *Ywhaz*. Mouse RT-PCR was performed to determine whether individual samples demonstrated upregulation with training, as was seen from the initial microarray analysis. Amplifications were performed in duplicate along with a no-DNA control for each sample condition, positive controls were added to each experiment. The TaqMan gene expression assay (Thermo Fisher, Waltham, MA, USA) was used for each gene: *Ywhaz* (Hs03044281_g1), *Card6* (Mm01297056_m1), *Impdh2* (Mm00496156_m1). The cycle threshold was determined for each tube, this was averaged between replicates and subtracted from that of the internal reference to get the delta cycle threshold (dCt) value. The difference between conditions was determined by calculating the differences between the dCt values to get the delta-delta cycle threshold (ddCt). An ANOVA compared 13 control/trained matched pairs. Fold change in gene expression was determined from the mean using the following equation: 2^(−ΔΔCt)^.

### 4.8. Human

Our blood samples originated from a study by Yiyuan Tang, comparing the effects of meditation to those of memory training on the brain, and were collected in accordance with Institutional Review Board approval at Texas Tech University. In his study, participants were randomly assigned a condition (meditation or memory) and were given training for a period of 2 weeks (personal communication). The meditation protocol is called Integrative Body–Mind Training (IBMT) and is a systemic training of attention and self-control while fostering acceptance and openness to the present experience [91]. The method does not promote controlling thought, but instead a develops state of restful alertness that allows awareness of body and breathing. Blood was extracted before and after training. We obtained samples from 26 healthy adults without any comorbidities (meditation: 8 males, 6 females, mean age 20.5 years; working memory: 7 males, 5 females, mean age 20.7 years). RNA was isolated using a Paxgene Blood RNA kit (Qiagen, Germantown, MD, USA) and evaluated via fragment analysis (Genomics and Cell Characterization Core Facility, UO, Eugene, OR, USA).

### 4.9. Real-Time PCR (Human)

As described above, individual samples were reverse transcribed and amplified using the TaqMan system, including the following gene probes: *Ywhaz* (Mm01158417_g1), *Card6* (Hs00261581_m1), *Impdh2* (Hs00168418_m1), *Dnase1l2* (Hs00357500_g1), *NF-κB2* (Hs01028901_g1), *Ikbkb* (Hs00233287_m1), and *Irf8* (Hs00175238_m1). Amplifications were performed in triplicate with a no-DNA control for each condition, along with positive controls in every experiment. Analysis was performed as previously described for mice. An ANOVA was used to compare changes in expression after to expression before by individual (within) and by group.

## 5. Conclusions

Overall, our study provides preliminary evidence that *Card6* is upregulated in mouse ACC, HC, and blood, and also in human blood, while *Impdh2* trended towards upregulation. Both of these genes we found, in common between the two species, to influence the same neural factor, NF-κB, that has been related both to innate and adaptive immunity, and learning. We also reported more detectable gene expression changes in the blood in response to our learning paradigm than in the two brain regions we studied. We believe that these results demonstrate that further examination is warranted of using blood as a proxy for genetic changes in the brain with learning. Gene set enrichment analysis also found evidence of upregulated genes related to collagen, which plays a role in the morphogenesis of neural tissue in the HC but not the ACC. Further research on mouse learning might yield additional insights into the molecular mechanisms of human learning and support efforts to improve learning and help in the case of disorders that diminish attention and memory.

## Figures and Tables

**Table 1:** Rank scores for the genes upregulated in mouse ACC, HC, and blood.

Gene	ACC	HC	Blood	Annotated Function
*Card6*	2.97	2.43	2.19	Signal transduction modulation
*Impdh2*	2.08	2.26	2.18	Nucleotide biosynthesis, cell growth
*Dnase1l2*	3.34	3.72	2.04	DNA catabolism, programmed cell death

**Table 2:** Combined average microarray intensity data for selected genes in mouse blood (log2 intensity values). Greater values indicate higher expression levels.

Gene	Pre, Control	Post, Control	Pre, Trained	Post, Trained
*Card6*	2.60	1.57	2.89	2.19
*Impdh2*	1.32	1.99	1.92	1.79
*Dnase1l2*	1.22	1.14	1.14	1.40
*Irf8*	4.12	3.31	3.93	3.86
*Ikbkb*	4.01	3.35	3.74	3.62
*NF-κB2*	2.95	2.58	3.22	2.82
*Ywhaz* (reference)	3.93	3.99	3.93	3.97

**Table 3:** Changes in gene expression in human blood following memory or meditation training (a negative number represents upregulation). These tables contain delta-delta Cycle Threshold (ddCt) values showing the difference between transcript levels in human blood before and after training.

Meditation (*n* = 14)				Memory (*n* = 12)			
Sample	*Card6*	*Impdh2*	*Dnase1l2*	Sample	*Card6*	*Impdh2*	*Dnase1l2*
11	−0.482	−1.616	0.422	1	−1.320	−0.833	0.255
13	−0.064	−0.197	0.279	2	−0.026	0.315	0.391
14	−0.766	0.191	0.113	3	−0.898	−0.010	−0.020
15	−0.014	−0.934	−0.470	4	−1.366	0.757	−1.100
16	−1.339	−0.510	0.176	6	0.038	−0.855	−0.407
17	−0.542	−0.748	−0.566	7	0.518	−0.736	−0.319
18	−0.231	−0.791	−0.228	8	0.508	−0.314	−0.851
19	−0.158	−0.754	0.135	9	−0.229	−0.448	−0.101
20	−0.081	0.021	−0.227	29	−1.391	0.004	−0.006
22	−1.241	0.906	0.060	30	−0.155	−0.057	−0.228
23	−0.585	0.471	0.432	31	0.119	−0.230	0.467
25	−1.968	0.489	0.229	32	−0.658	0.117	−0.053
26	−0.701	−0.125	−0.474				
27	−0.003	−0.244	0.242				
Mean	−0.584	−0.274	0.009	Mean	−0.405	−0.191	−0.164
SD	0.586	0.672	0.338	SD	0.703	0.484	0.465
Fold change	1.50×	1.21×	0.99×	Fold change	1.32×	1.14×	1.12×

**Table 4:** Summary of gene expression (mean delta-delta Cycle Threshold values), SD, and fold change for additional genes following memory or meditation training (a negative number represents upregulation).

Gene	*Irf8*		*NF-κB2*		*Ikbkb*	
Condition	Memory	Meditation	Memory	Meditation	Memory	Meditation
Mean	−0.308	0.655	−0.181	0.562	−0.270	0.161
SD	1.159	0.992	0.491	0.469	0.505	0.380
fold change	1.24×	0.64×	1.13×	0.68×	1.21×	0.89×

## Data Availability

The data presented in this study are openly available in NCBI’s Gene Expression Omnibus and are accessible through GEO Series accession number GSE244395 (https://www.ncbi.nlm.nih.gov/geo/query/acc.cgi?acc=GSE244395, accessed on 15 October 2023).

## Supplementary Materials

The following supporting information can be downloaded at https://www.mdpi.com/article/10.3390/ijms242115840/s1.

## References

[1] Barth A.L., Ray A. (2019). Progressive Circuit Changes during Learning and Disease. Neuron.

[2] Amoah D.K. (2022). Advances in the understanding and enhancement of the human cognitive functions of learning and memory. Brain Sci. Adv..

[3] Liu Q., Wu Y., Wang H., Jia F., Xu F. (2022). Viral Tools for Neural Circuit Tracing. Neurosci. Bull..

[4] Saunders A., Macosko E.Z., Wysoker A., Goldman M., Krienen F.M., de Rivera H., Bien E., Baum M., Bortolin L., Wang S. (2018). Molecular Diversity and Specializations among the Cells of the Adult Mouse Brain. Cell.

[5] Posner M.I., Sheese B.E., Odludaş Y., Tang Y. (2006). Analyzing and shaping human attentional networks. Neural Netw..

[6] Cullen C.L., Pepper R.E., Clutterbuck M.T., Pitman K.A., Oorschot V., Auderset L., Tang A.D., Ramm G., Emery B., Rodger J. (2021). Periaxonal and nodal plasticities modulate action potential conduction in the adult mouse brain. Cell Rep..

[7] Xin W., Chan J.R. (2020). Myelin plasticity: Sculpting circuits in learning and memory. Nat. Rev. Neurosci..

[8] Tang Y., Posner M.I. (2002). Attention training and Attention State Training. Trends Cogn. Sci..

[9] Melby-Lervåg M., Hulme C. (2013). Is working memory training effective? A meta-analytic review. Dev. Psychol..

[10] Tang Y.Y., Holzel B.K., Posner M.I. (2015). The neuroscience of mindfulness meditation. Nat. Rev. Neurosci..

[11] Posner M.I., Weible A.P., Voelker P., Rothbart M.K., Niell C.M. (2022). Decision Making as a Learned Skill in Mice and Humans. Front. Neurosci..

[12] Weible A.P. (2013). Remembering to Attend: The Anterior Cingulate Cortex and Remote Memory. Behav. Brain Res..

[13] Xu W., Südhof T.C. (2013). A Neural Circuit for Memory Specificity and Generalization. Science.

[14] Anderson M.C., Ochsner K.N., Kuhl B., Cooper J., Robertson E., Gabrieli S.W., Glover G.H., Gabrieli J.D.E. (2004). Neural Systems Underlying the Suppression of Unwanted Memories. Science.

[15] Bowman C.R., Zeithamova D. (2018). Abstract Memory Representations in the Ventromedial Prefrontal Cortex and Hippocampus Support Concept Generalization. J. Neurosci..

[16] Ciaramelli E., Grady C., Levine B., Ween J., Moscovitch M. (2010). Top-Down and Bottom-Up Attention to Memory Are Dissociated in Posterior Parietal Cortex: Neuroimaging and Neuropsychological Evidence. J. Neurosci..

[17] Weible A.P., Posner M.I., Neill C.M. (2019). Differential involvement of three brain regions during mouse skill learning. eNeuro.

[18] Tang Y.Y., Posner M.I. (2014). Training brain networks and states. Trends Cogn. Sci..

[19] Tang Y.Y., Lu Q., Geng X., Stein E.A., Yang Y., Posner M.I. (2010). Short-term meditation induces white matter changes in the anterior cingulate. Proc. Natl. Acad. Sci. USA.

[20] Tang Y.Y., Ma Y., Wang J., Fan Y., Feng S., Lu Q., Yu Q., Sui D., Rothbart M.K., Fan M. (2007). Short term meditation training improves attention and self-regulation. Proc. Natl. Acad. Sci. USA.

[21] Sánchez-Pérez N., Inuggi A., Castillo A., Campoy G., García-Santos J.M., González-Salinas C., Fuentes L.J. (2019). Computer-Based Cognitive Training Improves Brain Functional Connectivity in the Attentional Networks: A Study with Primary School-Aged Children. Front. Behav. Neurosci..

[22] Román F.J., Iturria-Medina Y., Martínez K., Karama S., Burgaleta M., Evans A.C., Jaeggi S.M., Colom R. (2017). Enhanced structural connectivity within a brain sub-network supporting working memory and engagement processes after cognitive training. Neurobiol. Learn. Mem..

[23] Bontempi B., Laurent-Demir C., Destrade C., Jaffard R. (1999). Time-dependent reorganization of brain circuitry underlying long-term memory storage. Nature.

[24] Frankland P.W., Bontempi B., Talton L.E., Kaczmarek L., Silva A.J. (2004). The involvement of the anterior cingulate cortex in remote contextual fear memory. Science.

[25] Rajasethupathy P., Sankaran S., Marshel J.H., Kim C.K., Ferenczi E., Lee S.Y., Berndt A., Ramakrishnan C., Jaffe A., Lo M. (2015). Projections from neocortex mediate top-down control of memory retrieval. Nature.

[26] Zhang Y., Fukushima H., Kida S. (2011). Induction and requirement of gene expression in the anterior cingulate cortex and medial prefrontal cortex for the consolidation of inhibitory avoidance memory. Mol. Brain.

[27] Tanimizu T., Kenney J.W., Okano E., Kadoma K., Frankland P.W., Kida S. (2017). Functional Connectivity of Multiple Brain Regions Required for the Consolidation of Social Recognition Memory. J. Neurosci..

[28] Nadler J.J., Zou F., Huang H., Moy S.S., Lauder J., Crawley J.N., Threadgill D.W., Wright F.A., Magnuson T.R. (2006). Large-scale gene expression differences across brain regions and inbred strains correlate with a behavioral phenotype. Genetics.

[29] Cai C., Langfelder P., Fuller T.F., Oldham M.C., Luo R., van den Berg L.H., Ophoff R.A., Horvath S. (2010). Is human blood a good surrogate for brain tissue in transcriptional studies?. BMC Genom..

[30] Harris S.E., Cox S.R., Bell S., Marioni R.E., Prins B.P., Pattie A., Corley J., Muñoz Maniega S., Valdés Hernández M., Morris Z. (2020). Eurology-related protein biomarkers are associated with cognitive ability and brain volume in older age. Nat. Commun..

[31] Vrillon A., Mouton-Liger F., Martinet M., Cognate E., Hourregue C., Dumurgier J., Bouaziz-Amar E., Brinkmalm A., Blennow K., Zetterberg H. (2022). Plasma neuregulin 1 as a synaptic biomarker in Alzheimer’s disease: A discovery cohort study. Alzheimer’s Res. Ther..

[32] McPhee G.M., Downey L.A., Stough C. (2020). Neurotrophins as a reliable biomarker for brain function, structure and cognition: A systematic review and meta-analysis. Neurobiol. Learn. Mem..

[33] Jones A., Jarvis P. (2017). Review of the potential use of blood neuro-biomarkers in the diagnosis of mild traumatic brain injury. Clin. Exp. Emerg. Med..

[34] Segura M., Pedreño C., Obiols J., Taurines R., Pàmias M., Grünblatt E., Gella A. (2015). Neurotrophin blood-based gene expression and social cognition analysis in patients with autism spectrum disorder. Neurogens.

[35] Santiago J.A., Bottero V., Potashkin J.A. (2018). Evaluation of RNA Blood Biomarkers in the Parkinson’s Disease Biomarkers Program. Front. Aging Neurosci..

[36] Atif H., Hicks S.D. (2019). A Review of MicroRNA Biomarkers in Traumatic Brain Injury. J. Exp. Neurosci..

[37] Ma G., Liu M., Du K., Zhong X., Gong S., Jiao L., Wei M. (2019). Differential Expression of mRNAs in the Brain Tissues of Patients with Alzheimer’s Disease Based on GEO Expression Profile and Its Clinical Significance. BioMed Res. Int..

[38] Milanesi E., Cucos C.A., Matias-Guiu J.A., Piñol-Ripoll G., Manda G., Dobre M., Cuadrado A. (2021). Reduced Blood RGS2 Expression in Mild Cognitive Impairment Patients. Front. Aging Neurosci..

[39] Pérez-González M., Badesso S., Lorenzo E., Guruceaga E., Pérez-Mediavilla A., García-Osta A., Cuadrado-Tejedor M. (2021). Identifying the Main Functional Pathways Associated with Cognitive Resilience to Alzheimer’s Disease. Int. J. Mol. Sci..

[40] Klein A.B., Williamson R., Santini M.A., Clemmensen C., Ettrup A., Rios M., Knudsen G.M., Aznar S. (2011). Blood BDNF concentrations reflect brain-tissue BDNF levels across species. Int. J. Neuropsychopharmacol..

[41] Amagase Y., Kambayashi R., Sugiyama A., Takei Y. (2023). Peripheral Regulation of Central Brain-Derived Neurotrophic Factor Expression through the Vagus Nerve. Int. J. Mol. Sci..

[42] Subramanian A., Tamayo P., Mootha V.K., Mukherjee S., Ebert B.L., Gillette M.A., Paulovich A., Pomeroy S.L., Golub T.R., Lander E.S. (2005). Gene set enrichment analysis: A knowledge-based approach for interpreting genome-wide expression profiles. Proc. Natl. Acad. Sci. USA.

[43] Dufner A., Pownall S., Mak T.W. (2006). Caspase recruitment domain protein 6 is a microtubule-interacting protein that positively modulates NF-kappaB activation. Proc. Natl. Acad. Sci. USA.

[44] Toubiana J., Rossi A.L., Grimaldi D., Belaidouni N., Chafey P., Clary G., Courtine E., Pene F., Mira J.P., Claessens Y.E. (2011). IMPDHII protein inhibits Toll-like receptor 2-mediated activation of NF-kappaB. J. Biol. Chem..

[45] Shiokawa D., Matsushita T., Kobayashi T., Matsumoto Y., Tanuma S. (2004). Characterization of the human DNAS1L2 gene and the molecular mechanism for its transcriptional activation induced by inflammatory cytokines. Genomics.

[46] Vijayendran C., Bermudez M.-L., Koka M., Chandran B., Pawale D., Vishnubhotla R., Alankar S., Maturi R., Subramaniam B., Sadhasivam S. (2021). Large-scale genomic study reveals robust activation of the immune system following advanced Inner Engineering meditation retreat. Proc. Natl. Acad. Sci. USA.

[47] Bonefeld B.E., Elfving B., Wegener G. (2008). Reference genes for normalization: A study of rat brain tissue. Synapse.

[48] Gladkikh A., Potashnikova D., Korneva E., Khudoleeva O., Vorobjev I. (2010). Cyclin D1 expression in B-cell lymphomas. Exp. Hematol..

[49] Yan Z., Gao J., Lv X., Yang W., Wen S., Tong H., Tang C. (2016). Quantitative Evaluation and Selection of Reference Genes for Quantitative RT-PCR in Mouse Acute Pancreatitis. BioMed Res. Int..

[50] Yousif A., Drou N., Rowe J., Khalfan M., Gunsalus K.C. (2020). NASQAR: A web-based platform for high-throughput sequencing data analysis and visualization. BMC Bioinform..

[51] Capece D., Verzella D., Flati I., Arboreta P., Cornice J., Franzoso G. (2022). NF-κB: Blending metabolism, immunity, and inflammation. Trends Immunol..

[52] Calabria E., Mazza E.M., Dyar K.A., Pogliaghi S., Bruseghini P., Morandi C., Salvagno G.L., Gelati M., Guidi G.C., Bicciato S. (2016). Aging: A portrait from gene expression profile in blood cells. Aging.

[53] Jaeggi S.M., Buschkuehl M., Jonides J., Perrig W.J. (2008). Improving fluid intelligence with training on working memory. Proc. Natl. Acad. Sci. USA.

[54] Tang Y.Y., Ma Y., Fan Y., Feng H., Wang J., Feng S., Lu Q., Hu B., Lin Y., Li J. (2009). Central and autonomic nervous system interaction is altered by short-term meditation. Proc. Natl. Acad. Sci. USA.

[55] Long K.R., Husttner W.B. (2019). How the extracellular matrix shapes neural development. Open Biol..

[56] Chernousov M.A., Rothblum K., Stahl R.C., Evans A., Prentiss L., Carey D.J. (2006). Glypican-1 and alpha4(V) collagen are required for Schwann cell myelination. J. Neurosci..

[57] Hubert T., Grimal S., Carroll P., Fichard-Carroll A. (2009). Collagens in the developing and diseased nervous system. Cell Mol. Life Sci..

[58] Fox M.A. (2008). Novel roles for collagens in wiring the vertebrate nervous system. Curr. Opin. Cell Biol..

[59] Kakoi C., Udo H., Matsukawa T., Ohnuki K. (2012). Collagen peptides enhance hippocampal neurogenesis and reduce anxiety related behavior in mice. Biomed. Res..

[60] Shin S.S., Grandhi R., Henchir J., Yan H.Q., Badylak S.F., Dixon C.E. (2015). Neuroprotective effects of collagen matrix in rats after traumatic brain injury. Restor. Neurol. Neurosci..

[61] Stehlik C., Hayashi H., Pio F., Godzik A., Reed J.C. (2003). Card6 is a modulator of NF-kappa B activation by Nod1- and Cardiak-mediated pathways. J. Biol. Chem..

[62] Zhao M., He H., Yin J. (2020). Card6 protects against collagen-induced rheumatoid arthritis in mice through attenuating the inflammatory response and joint destruction via suppression of TNFR1/TRAF2 signaling. Biochem. Biophys. Res. Commun..

[63] Wang J.L., Luo X., Liu L. (2019). Targeting Card6 attenuates spinal cord injury (SCI) in mice through inhibiting apoptosis, inflammation and oxidative stress associated ROS production. Aging.

[64] Weber G., Nakamura H., Natsumeda Y., Szekeres T., Nagai M. (1992). Regulation of GTP biosynthesis. Adv. Enzym. Regul..

[65] Kuukasjärvi A., Landoni J.C., Kaukonen J., Juhakoski M., Auranen M., Torkkeli T., Velagapudi V., Suomalainen A. (2021). IMPDH2: A new gene associated with dominant juvenile-onset dystonia-tremor disorder. Eur. J. Hum. Genet..

[66] Zanon A., Rakovic A., Blankenburg H., Doncheva N.T., Schwienbacher C., Serafin A., Alexa A., Weichenberger C.X., Albrecht M., Klein C. (2013). Profiling of Parkin-binding partners using tandem affinity purification. PLoS ONE.

[67] Li T.W., Kenney A.D., Park J.G., Fiches G.N., Liu H., Zhou D., Biswas A., Zhao W., Que J., Santoso N. (2022). SARS-CoV-2 Nsp14 protein associates with IMPDH2 and activates NF-κB signaling. Front. Immunol..

[68] Liao L.X., Song X.M., Wang L.C., Lv H.N., Chen J.F., Liu D., Fu G., Zhao M.B., Jiang Y., Zeng K.W. (2017). Highly selective inhibition of IMPDH2 provides the basis of anti-neuroinflammation therapy. Proc. Natl. Acad. Sci. USA.

[69] https://maayanlab.cloud/Harmonizome/gene_set/NF-KappaB/MotifMap+Predicted+Transcription+Factor+Targets.

[70] Brown G.R., Hem V., Katz K.S., Ovetsky M., Wallin C., Ermolaeva O., Tolstoy I., Tatusova T., Pruitt K.D., Maglott D.R. (2015). Gene: A gene-centered information resource at NCBI. Nucleic Acids Res..

[71] Kaltschmidt B., Kaltschmidt C. (2015). NF-KappaB in long-term memory and structural plasticity in the adult mammalian brain. Front. Mol. Neurosci..

[72] Alberini C.M. (2009). Transcription Factors in Long-Term Memory and Synaptic Plasticity. Physiol. Rev..

[73] Marin I., Kipnis J. (2013). Learning and memory... and the immune system. Learn. Mem..

[74] Alani B., Salehi R., Sadeghi P., Zare M., Khodagholi F., Arefian E., Hakemi M.G., Digaleh H. (2014). Silencing of Hsp90 chaperone expression protects against 6-hydroxydopamine toxicity in PC12 cells. J. Mol. Neurosci..

[75] Sanati M., Aminyavari S., Khodagholi F., Hajipour M.J., Sadeghi P., Noruzi M., Moshtagh A., Behmadi H., Sharifzadeh M. (2021). PEGylated superparamagnetic iron oxide nanoparticles (SPIONs) ameliorate learning and memory deficit in a rat model of Alzheimer’s disease: Potential participation of STIMs. Neurotox.

[76] Wardyn J.D., Ponsford A.H., Sanderson C.M. (2015). Dissecting molecular cross-talk between Nrf2 and NF-κB response pathways. Biochem. Soc. Trans..

[77] Barnabei L., Laplantine E., Mbongo W., Rieux-Laucat F., Weil R. (2021). NF-κB: At the Borders of Autoimmunity and Inflammation. Front. Immunol..

[78] Filiano A.J., Gadani S.P., Kipnis J. (2015). Interactions of innate and adaptive immunity in brain development and function. Brain Res..

[79] Zhao F., Li B., Yang W., Ge T., Cui R. (2022). Brain-immune interaction mechanisms: Implications for cognitive dysfunction in psychiatric disorders. Cell Prolif..

[80] Werneburg S., Feinberg P.A., Johnson K.M., Schafer D.P. (2017). A microglia-cytokine axis to modulate synaptic connectivity and function. Curr. Opin. Neurobiol..

[81] Brynskikh A., Warren T., Zhu J., Kipnis J. (2008). Adaptive immunity affects learning behavior in mice. Brain Behav. Immun..

[82] Ziv Y., Schwartz M. (2008). Immune-based regulation of adult neurogenesis: Implications for learning and memory. Brain Behav. Immun..

[83] Wada Y. (2013). Vacuoles in mammals: A subcellular structure indispensable for early embryogenesis. Bioarchitecture.

[84] Qi C., Luo L.D., Feng I., Ma S. (2022). Molecular mechanisms of synaptogenesis. Front. Synaptic Neurosci..

[85] Batool A., Cheng J., Liu Y.-X. (2019). Role of EZH2 in cell lineage determination and relative signaling pathways. Front. Biosci..

[86] Bilbo S.D., Schwarz J.M. (2012). The immune system and developmental programming of brain and behavior. Front. Neuroendocrinol..

[87] Dantzer R., Cohen S., Russo S.J., Dinan T.G. (2018). Resilience and immunity. Brain Behav. Immun..

[88] Rollins B., Martin M.V., Morgan L., Vawter M.P. (2010). Analysis of whole genome biomarker expression in blood and brain. Am. J. Med. Genet. Part B Neuropsychiatr. Genet..

[89] Irizarry R.A., Hobbs B., Collin F., Beazer-Barclay Y.D., Antonellis K.J., Scherf U., Speed T.P. (2003). Exploration, normalization, and summaries of high density oligonucleotide array probe level data. Biostats.

[90] Dai M., Wang P., Boyd A.D., Kostov G., Athey B., Jones E.G., Bunney W.E., Myers R.M., Speed T.P., Akil H. (2005). Evolving gene/transcript definitions significantly alter the interpretation of GeneChip data. Nucleic Acids Res..

[91] Tang Y.Y., Tang R., Gross J.J. (2019). Promoting Psychological Well-Being Through an Evidence-Based Mindfulness Training Program. Front. Hum. Neurosci..

