# Supplementary material for: Molecular Mechanisms for Changing Brain Connectivity in Mice and Humans
Source: Int J Mol Sci. 2023 Oct 31;24(21):15840. doi: 10.3390/ijms242115840 (PMC10648558; doi:10.3390/ijms242115840)
Supplement: Supplementary file 1 [file ijms-24-15840-s001.zip › ijms-2666007-supplementary.pdf]

Appendix

Table S1. The rank scores of 25 genes in common that are upregulated with training

		rank scores	
gene abbreviation	full gene name	ACC	HC
astl	astacin like metalloendopeptidase	2.9782302	2.6145406
c9	complement C9	2.4242568	2.9751272
cacna2d4	calcium voltage-gated channel auxiliary subunit alpha2delta 4	2.5569508	3.471862
card6	caspase recruitment domain family member 6	2.9706614	2.433023
cd40	CD40 molecule	2.4512355	3.8273673
dmrtb1	DMRT like family B with proline rich C-terminal 1	2.6347992	3.5000591
dnai2	dynein axonemal intermediate chain 2	2.026856	3.2761416
dnase1l2	deoxyribonuclease 1 like 2	3.3436997	3.72264
entpd8	ectonucleoside triphosphate diphosphohydrolase 8	2.0840733	2.364991
ghrh	growth hormone releasing hormone	2.8068497	2.369232
hmox1	heme oxygenase 1	2.5849724	2.5780656
impdh2	inosine monophosphate dehydrogenase 2	2.0824804	2.2658856
inhbb	inhibin beta B subunit	2.61479	2.8082063
mbnl3	muscleblind like splicing regulator 3	3.1857593	2.489401
mnx1	motor neuron and pancreas homeobox 1	3.6334696	4.335227
nkx6-1	NK6 homeobox 1	2.2450445	3.0883238
prr15l	proline rich 15 like	2.5259113	4.2262025
rps7	ribosomal protein S7	3.7486062	3.9771392
senp8	SUMO/sentrin peptidase family member, NEDD8 specific	3.3826416	2.3196073
sftpa1	surfactant protein A1	2.1839666	5.2199636
skp2	S-phase kinase associated protein 2	2.2981544	3.0969088
steap3	STEAP3 metalloreductase	2.4964416	2.7268634
tbx5	T-box 5	2.2913268	3.3157961
trim7	tripartite motif containing 7	2.114829	2.5109558
znf217	zinc finger protein 217	2.3345547	2.2881632

Table S2. The GSEA results in mouse brain showing the effect of region x training. (NES, normalized enrichment score; p, significance; FDR q, significance when corrected for multiple testing).

	**region:training**			**ACC, trained vs control**			**HC, trained vs control**		
**Gene Set Name**	**NES**	***p***	**FDR *q***	**NES**	***p***	**FDR *q***	**NES**	***p***	**FDR *q***
REACTOME_COLLAGEN_CHAIN_TRIMERIZATION	2.35	0.0000	***0.0000***	-1.96	0.0000	***0.1332***	1.87	0.0000	***0.2134***
HALLMARK_PROTEIN_SECRETION	-2.34	0.0000	***0.0000***	1.78	0.0000	0.4323	-1.78	0.0000	0.2595
GOBP_TRANSEPITHELIAL_TRANSPORT	2.25	0.0000	***0.0050***	-1.82	0.0000	0.3015	1.62	0.0151	0.3531
GOMF_EXTRACELLULAR_MATRIX_STRUCTURAL_CONSTITUENT_CONFERRING_TENSILE_STRENGTH	2.25	0.0000	***0.0053***	-2.00	0.0000	***0.1305***	1.85	0.0000	***0.2265***
REACTOME_COLLAGEN_DEGRADATION	2.15	0.0000	***0.0227***	-2.00	0.0000	***0.1740***	1.68	0.0044	0.2923
REACTOME_ASSEMBLY_OF_COLLAGEN_FIBRILS_AND_OTHER_MULTIMERIC_STRUCTURES	2.13	0.0000	***0.0237***	-2.13	0.0000	***0.0744***	1.92	0.0000	***0.1715***

Table 3. The GSEA results in mouse blood showing the effect of training. (NES, normalized enrichment score; p, significance; FDR q, significance when corrected for multiple testing).

**Group**	**Gene Set Name**	**NES**	***p***	**FDR *q***
		**post, trained vs control**		
C2 WikiPathways pathway	WP_B_CELL_RECEPTOR_SIGNALING_PATHWAY	2.51	0.0000	***0.0000***
Hallmark	HALLMARK_OXIDATIVE_PHOSPHORYLATION	2.32	0.0000	***0.0000***
C2 PID pathway	PID_BCR_5PATHWAY	2.42	0.0000	***0.0000***
C2 KEGG pathway	KEGG_B_CELL_RECEPTOR_SIGNALING_PATHWAY	2.37	0.0000	***0.0000***
C5 GO Biological Process	GOBP_ANTIGEN_RECEPTOR_MEDIATED_SIGNALING_PATHWAY	2.33	0.0000	***0.0000***
C5 GO Biological Process	GOBP_GOLGI_VESICLE_TRANSPORT	2.29	0.0000	***0.0002***
C2 Reactome pathway	REACTOME_SIGNALING_BY_THE_B_CELL_RECEPTOR_BCR	2.27	0.0000	***0.0005***
C5 GO Biological Process	GOBP_B_CELL_RECEPTOR_SIGNALING_PATHWAY	2.25	0.0000	***0.0009***
C2 PID pathway	PID_ATM_PATHWAY	2.25	0.0000	***0.0010***
C2 Reactome pathway	REACTOME_TCR_SIGNALING	2.23	0.0000	***0.0012***
C2 PID pathway	PID_TNF_PATHWAY	2.22	0.0000	***0.0014***
Hallmark	HALLMARK_ALLOGRAFT_REJECTION	2.19	0.0000	***0.0017***
C5 GO Biological Process	GOBP_INTRA_GOLGI_VESICLE_MEDIATED_TRANSPORT	2.19	0.0000	***0.0018***
C2 WikiPathways pathway	WP_IL3_SIGNALING_PATHWAY	2.19	0.0000	***0.0019***
C2 Reactome pathway	REACTOME_HIV_INFECTION	2.19	0.0000	***0.0021***
Hallmark	HALLMARK_MTORC1_SIGNALING	2.19	0.0000	***0.0021***
C2 Reactome pathway	REACTOME_INTRA_GOLGI_TRAFFIC	2.20	0.0000	***0.0022***
C2 Reactome pathway	REACTOME_TRANSLOCATION_OF_SLC2A4_GLUT4_TO_THE_PLASMA_MEMBRANE	2.19	0.0000	***0.0022***
C5 GO Biological Process	GOBP_IMMUNE_RESPONSE_REGULATING_CELL_SURFACE_RECEPTOR_SIGNALING_PATHWAY	2.17	0.0000	***0.0026***
C5 GO Biological Process	GOBP_T_CELL_RECEPTOR_SIGNALING_PATHWAY	2.17	0.0000	***0.0027***
C2 Reactome pathway	REACTOME_SIGNALING_BY_TGF_BETA_RECEPTOR_COMPLEX	2.16	0.0000	***0.0032***
C2 Reactome pathway	REACTOME_ER_TO_GOLGI_ANTEROGRADE_TRANSPORT	2.15	0.0000	***0.0034***
C2 PID pathway	PID_TCR_PATHWAY	2.14	0.0000	***0.0037***
C2 Reactome pathway	REACTOME_GENERATION_OF_SECOND_MESSENGER_MOLECULES	2.14	0.0000	***0.0038***
C5 GO Cellular Component	GOCC_ENDOPLASMIC_RETICULUM_PROTEIN_CONTAINING_COMPLEX	2.14	0.0000	***0.0039***

C5 GO Cellular Component	GOCC_FICOLIN_1_RICH_GRANULE	2.14	0.0000	***0.0039***
C5 GO Biological Process	GOBP_IMMUNE_RESPONSE_REGULATING_SIGNALING_PATHWAY	2.13	0.0000	***0.0039***
C2 Reactome pathway	REACTOME_FC_EPSILON_RECEPTOR_FCERI_SIGNALING	2.13	0.0000	***0.0040***
C2 WikiPathways pathway	WP_STEROL_REGULATORY_ELEMENTBINDING_PROTEINS_SREBP_SIGNALING	2.13	0.0000	***0.0041***
C2 Reactome pathway	REACTOME_FORMATION_OF_TUBULIN_FOLDING_INTERMEDIATES_BY_CCT_TRIC	2.13	0.0000	***0.0041***
C2 Reactome pathway	REACTOME_INTRA_GOLGI_AND_RETROGRADE_GOLGI_TO_ER_TRAFFIC	2.12	0.0000	***0.0042***
C2 PID pathway	PID_RAC1_PATHWAY	2.11	0.0000	***0.0043***
C5 GO Cellular Component	GOCC_FICOLIN_1_RICH_GRANULE_LUMEN	2.10	0.0000	***0.0043***
C5 GO Biological Process	GOBP_GOLGI_TO_VACUOLE_TRANSPORT	2.11	0.0000	***0.0044***
Hallmark	HALLMARK_MYC_TARGETS_V1	2.12	0.0000	***0.0044***
C2 Reactome pathway	REACTOME_NEUTROPHIL_DEGRANULATION	2.09	0.0000	***0.0044***
C5 GO Biological Process	GOBP_RETROGRADE_VESICLE_MEDIATED_TRANSPORT_GOLGI_TO_ENDOPLASMIC_RETICULUM	2.10	0.0000	***0.0044***
C2 KEGG pathway	KEGG_NATURAL_KILLER_CELL_MEDIATED_CYTOTOXICITY	2.11	0.0000	***0.0044***
C2 Reactome pathway	REACTOME_HOST_INTERACTIONS_OF_HIV_FACTORS	2.10	0.0000	***0.0044***
C2 Reactome pathway	REACTOME_G2_M_CHECKPOINTS	2.10	0.0000	***0.0045***
C2 Reactome pathway	REACTOME_DEUBIQUITINATION	2.10	0.0000	***0.0045***
C2 Reactome pathway	REACTOME_TNF_SIGNALING	2.09	0.0000	***0.0046***

**Group**	**Gene Set Name**	**NES**	***p***	**FDR *q***
		**post, trained vs control**		
C5 GO Biological Process	GOBP_NEGATIVE_REGULATION_OF_RESPONSE_TO_ENDOPLASMIC_RETICULUM_STRESS	2.08	0.0000	***0.0050***
C5 GO Biological Process	GOBP_CELLULAR_RESPIRATION	2.08	0.0000	***0.0051***
C2 Reactome pathway	REACTOME_INTERLEUKIN_3_INTERLEUKIN_5_AND_GM_CSF_SIGNALING	2.08	0.0000	***0.0051***
C2 WikiPathways pathway	WP_MODULATORS_OF_TCR_SIGNALING_AND_T_CELL_ACTIVATION	2.08	0.0000	***0.0052***
C2 PID pathway	PID_PDGFRB_PATHWAY	2.08	0.0000	***0.0052***
C2 Reactome pathway	REACTOME_CLEC7A_DECTIN_1_SIGNALING	2.08	0.0000	***0.0052***
C2 Reactome pathway	REACTOME_COSTIMULATION_BY_THE_CD28_FAMILY	2.07	0.0000	***0.0053***
C2 Reactome pathway	REACTOME_ANTIGEN_ACTIVATES_B_CELL_RECEPTOR_BCR_LEADING_TO_GENERATION_OF_SECOND_MESSENGERS	2.07	0.0000	***0.0053***
C2 PID pathway	PID_FAS_PATHWAY	2.07	0.0000	***0.0054***
C5 GO Cellular Component	GOCC_VESICLE_TETHERING_COMPLEX	2.07	0.0000	***0.0054***

C2 KEGG pathway	KEGG_SPLICEOSOME	2.07	0.0000	***0.0054***
C5 GO Biological Process	GOBP_NATURAL_KILLER_CELL_MEDIATED_IMMUNITY	2.06	0.0000	***0.0054***
C2 WikiPathways pathway	WP_TNFALPHA_SIGNALING_PATHWAY	2.07	0.0000	***0.0054***
C5 GO Biological Process	GOBP_MEMBRANE_RAFT_ORGANIZATION	2.06	0.0000	***0.0055***
C2 Reactome pathway	REACTOME_ANTIGEN_PROCESSING_CROSS_PRESENTATION	2.06	0.0000	***0.0055***
C5 GO Biological Process	GOBP_POST_GOLGI_VESICLE_MEDIATED_TRANSPORT	2.07	0.0000	***0.0055***
C2 KEGG pathway	KEGG_FC_GAMMA_R_MEDIATED_PHAGOCYTOSIS	2.06	0.0000	***0.0055***
C2 Reactome pathway	REACTOME_REGULATION_OF_TNFR1_SIGNALING	2.06	0.0000	***0.0055***
C5 GO Molecular Function	GOMF_PROTEIN_N_TERMINUS_BINDING	2.06	0.0000	***0.0055***
C2 PID pathway	PID_IL12_2PATHWAY	2.06	0.0000	***0.0056***
C2 Reactome pathway	REACTOME_COOPERATION_OF_PREFOLDIN_AND_TRIC_CCT_IN_ACTIN_AND_TUBULIN_FOLDING	2.06	0.0000	***0.0056***
C2 Reactome pathway	REACTOME_PROCESSING_OF_CAPPED_INTRON_CONTAINING_PRE_MRNA	2.05	0.0000	***0.0056***
C2 Reactome pathway	REACTOME_TRANSCRIPTIONAL_ACTIVITY_OF_SMAD2_SMAD3_SMAD4_HETEROTRIMER	2.05	0.0000	***0.0056***
C2 Reactome pathway	REACTOME_HSP90_CHAPERONE_CYCLE_FOR_STEROID_HORMONE_RECEPTORS_SHR_IN_THE_PRESENCE_OF_LIGAND	2.05	0.0000	***0.0057***
C2 Reactome pathway	REACTOME_COPI_INDEPENDENT_GOLGI_TO_ER_RETROGRADE_TRAFFIC	2.04	0.0000	***0.0064***
C2 WikiPathways pathway	WP_4HYDROXYTAMOXIFEN_DEXAMETHASONE_AND_RETINOIC_ACIDS_REGULATION_OF_P27_EXPRESSION	2.04	0.0000	***0.0064***
C2 WikiPathways pathway	WP_TCELL_ACTIVATION_SARSCOV2	2.04	0.0000	***0.0064***
C2 WikiPathways pathway	WP_TCELL_ANTIGEN_RECEPTOR_TCR_PATHWAY_DURING_STAPHYLOCOCCUS_AUREUS_INFECTION	2.04	0.0000	***0.0064***
C2 WikiPathways pathway	WP_INTERACTIONS_OF_NATURAL_KILLER_CELLS_IN_PANCREATIC_CANCER	2.04	0.0000	***0.0065***
C2 PID pathway	PID_P38_ALPHA_BETA_PATHWAY	2.04	0.0000	***0.0065***
C2 PID pathway	PID_CD8_TCR_PATHWAY	2.03	0.0000	***0.0066***
C2 PID pathway	PID_PI3KCI_PATHWAY	2.03	0.0000	***0.0068***
C2 Reactome pathway	REACTOME_PROTEIN_LOCALIZATION	2.03	0.0000	***0.0069***
C5 GO Cellular Component	GOCC_SPLICEOSOMAL_SNRNP_COMPLEX	2.03	0.0000	***0.0071***
C5 GO Cellular Component	GOCC_NUCLEAR_PERIPHERY	2.02	0.0000	***0.0073***
C2 KEGG pathway	KEGG_PATHOGENIC_ESCHERICHIA_COLI_INFECTION	2.02	0.0000	***0.0073***
C5 GO Biological Process	GOBP_NEGATIVE_REGULATION_OF_B_CELL_ACTIVATION	2.02	0.0000	***0.0075***
C5 GO Biological Process	GOBP_REGULATION_OF_TRANSLATIONAL_INITIATION	2.02	0.0000	***0.0076***
C2 Reactome pathway	REACTOME_TRANSCRIPTIONAL_REGULATION_BY_RUNX3	2.01	0.0000	***0.0079***

C5 GO Biological Process	GOBP_OXIDATIVE_PHOSPHORYLATION	2.01	0.0000	***0.0079***
C5 GO Biological Process	GOBP_RETROGRADE_TRANSPORT_ENDOSOME_TO_GOLGI	2.01	0.0000	***0.0081***
C2 WikiPathways pathway	WP_PATHOGENIC_ESCHERICHIA_COLI_INFECTION	2.01	0.0000	***0.0081***

Group	Gene Set Name	NES	*p*	FDR *q*
		**post, trained vs control**		
C2 WikiPathways pathway	WP_IL1_SIGNALING_PATHWAY	2.01	0.0000	***0.0083***
C2 WikiPathways pathway	WP_MRNA_PROCESSING	2.01	0.0000	***0.0084***
C2 Reactome pathway	REACTOME_TNFR1_INDUCED_NFKAPPAB_SIGNALING_PATHWAY	2.00	0.0000	***0.0086***
C2 Reactome pathway	REACTOME_CLASS_I_MHC_MEDIATED_ANTIGEN_PROCESSING_PRESENTATION	2.00	0.0000	***0.0088***
C5 GO Molecular Function	GOMF_RIBOSOME_BINDING	2.00	0.0000	***0.0089***
C2 Reactome pathway	REACTOME_G2_M_DNA_DAMAGE_CHECKPOINT	2.00	0.0000	***0.0089***
C2 Reactome pathway	REACTOME_SIGNALING_BY_NOTCH4	2.00	0.0000	***0.0090***
C2 WikiPathways pathway	WP_MICROGLIA_PATHOGEN_PHAGOCYTOSIS_PATHWAY	1.99	0.0000	***0.0090***
C5 GO Biological Process	GOBP_PROTEIN_MODIFICATION_BY_SMALL_PROTEIN_REMOVAL	1.99	0.0000	***0.0090***
C2 Reactome pathway	REACTOME_TRANSPORT_TO_THE_GOLGI_AND_SUBSEQUENT_MODIFICATION	2.00	0.0000	***0.0090***
C2 BioCarta pathway	BIOCARTA_PROTEASOME_PATHWAY	1.99	0.0019	***0.0091***
C5 GO Biological Process	GOBP_MACROAUTOPHAGY	1.99	0.0000	***0.0091***
C2 WikiPathways pathway	WP_HEPATITIS_B_INFECTION	1.99	0.0000	***0.0091***
C2 KEGG pathway	KEGG_TYPE_II_DIABETES_MELLITUS	1.99	0.0000	***0.0092***
C2 WikiPathways pathway	WP_TGFBETA_SIGNALING_PATHWAY	1.99	0.0000	***0.0092***
C5 GO Molecular Function	GOMF_SNORNA_BINDING	1.99	0.0018	***0.0092***
C2 BioCarta pathway	BIOCARTA_HCMV_PATHWAY	1.99	0.0000	***0.0092***
C2 WikiPathways pathway	WP_THYMIC_STROMAL_LYMPHOPOIETIN_TSLP_SIGNALING_PATHWAY	1.99	0.0000	***0.0092***
C2 KEGG pathway	KEGG_FC_EPSILON_RI_SIGNALING_PATHWAY	1.99	0.0000	***0.0092***
C5 GO Biological Process	GOBP_INTRINSIC_APOPTOTIC_SIGNALING_PATHWAY_IN_RESPONSE_TO_OXIDATIVE_STRESS	1.99	0.0000	***0.0093***
C5 GO Cellular Component	GOCC_MEDIATOR_COMPLEX	1.98	0.0000	***0.0094***
C2 Reactome pathway	REACTOME_S_PHASE	1.98	0.0000	***0.0094***
C5 GO Biological Process	GOBP_PROTEIN_LOCALIZATION_TO_CHROMOSOME	1.98	0.0000	***0.0095***
C5 GO Biological Process	GOBP_ENDOPLASMIC_RETICULUM_TO_GOLGI_VESICLE_MEDIATED_TRANSPORT	1.98	0.0000	***0.0096***

C2 Reactome pathway	REACTOME_DOWNSTREAM_SIGNALING_EVENTS_OF_B_CELL_RECEPTOR_BCR	1.98	0.0000	***0.0097***
C2 WikiPathways pathway	WP_IL5_SIGNALING_PATHWAY	1.98	0.0000	***0.0097***
C2 WikiPathways pathway	WP_LEPTIN_SIGNALING_PATHWAY	1.98	0.0018	***0.0099***
C5 GO Molecular Function	GOMF_RIBONUCLEOPROTEIN_COMPLEX_BINDING	1.98	0.0000	***0.0099***
C2 BioCarta pathway	BIOCARTA_FCER1_PATHWAY	1.97	0.0000	***0.0100***
C2 WikiPathways pathway	WP_TCELL_RECEPTOR_TCR_SIGNALING_PATHWAY	1.97	0.0000	***0.0100***
C2 WikiPathways pathway	WP_TYROBP_CAUSAL_NETWORK_IN_MICROGLIA	1.97	0.0000	***0.0100***
C5 GO Molecular Function	GOMF_SIGNAL_SEQUENCE_BINDING	1.97	0.0000	***0.0100***
C2 Reactome pathway	REACTOME_FCERI_MEDIATED_NF_KB_ACTIVATION	1.97	0.0000	***0.0101***
C2 Reactome pathway	REACTOME_COPI_MEDIATED_ANTEROGRADE_TRANSPORT	1.97	0.0000	***0.0102***
C2 WikiPathways pathway	WP_PROLACTIN_SIGNALING_PATHWAY	1.97	0.0000	***0.0103***
C5 GO Molecular Function	GOMF_ISOPEPTIDASE_ACTIVITY	1.97	0.0000	***0.0103***
C2 Reactome pathway	REACTOME_CRISTAE_FORMATION	1.97	0.0000	***0.0103***
C2 Reactome pathway	REACTOME_TRANSCRIPTIONAL_REGULATION_BY_RUNX1	1.97	0.0000	***0.0103***
C2 Reactome pathway	REACTOME_MYD88_INDEPENDENT_TLR4_CASCADE	1.97	0.0000	***0.0103***
C2 Reactome pathway	REACTOME_INFLAMMASOMES	1.96	0.0000	***0.0104***
C5 GO Molecular Function	GOMF_INOSITOL_PHOSPHATE_PHOSPHATASE_ACTIVITY	1.96	0.0000	***0.0104***
C5 GO Biological Process	GOBP_REGULATION_OF_RESPONSE_TO_ENDOPLASMIC_RETICULUM_STRESS	1.96	0.0000	***0.0104***

Group	Gene Set Name	NES	*p*	FDR *q*
		post, trained vs control		
C2 Reactome pathway	REACTOME_DOWNREGULATION_OF_SMAD2_3_SMAD4_TRANSCRIPTIONAL_ACTIVITY	1.96	0.0000	***0.0105***
C5 GO Biological Process	GOBP_ACTIVATION_OF_IMMUNE_RESPONSE	1.96	0.0000	***0.0107***
C2 BioCarta pathway	BIOCARTA_STRESS_PATHWAY	1.96	0.0000	***0.0108***
C2 Reactome pathway	REACTOME_DEATH_RECEPTOR_SIGNALLING	1.95	0.0000	***0.0110***
C5 GO Molecular Function	GOMF_NUCLEAR_RECEPTOR_COACTIVATOR_ACTIVITY	1.95	0.0000	***0.0110***
C2 Reactome pathway	REACTOME_FLT3_SIGNALING	1.95	0.0000	***0.0110***
C5 GO Biological Process	GOBP_T_CELL_ACTIVATION	1.95	0.0000	***0.0111***
Hallmark	HALLMARK_PI3K_AKT_MTOR_SIGNALING	1.96	0.0000	***0.0111***
C5 GO Molecular Function	GOMF_TRANSLATION_ELONGATION_FACTOR_ACTIVITY	1.95	0.0000	***0.0111***
C2 WikiPathways pathway	WP_GASTRIC_CANCER_NETWORK_2	1.95	0.0000	***0.0111***
C5 GO Biological Process	GOBP_LEUKOCYTE_DEGRANULATION	1.95	0.0000	***0.0111***
C5 GO Biological Process	GOBP_NUCLEOLAR_LARGE_RRNA_TRANSCRIPTION_BY_RNA_POLYMERASE_I	1.95	0.0000	***0.0111***

C5 GO Biological Process	GOBP_MEMBRANE_LIPID_CATABOLIC_PROCESS	1.95	0.0000	***0.0112***
C2 Reactome pathway	REACTOME_METALLOPROTEASE_DUBS	1.95	0.0000	***0.0112***
C5 GO Biological Process	GOBP_LYMPHOCYTE_COSTIMULATION	1.95	0.0019	***0.0112***
C2 Reactome pathway	REACTOME_ASPARAGINE_N_LINKED_GLYCOSYLATION	1.95	0.0000	***0.0113***
C2 KEGG pathway	KEGG_CHRONIC_MYELOID_LEUKEMIA	1.95	0.0000	***0.0113***
C2 Reactome pathway	REACTOME_TRANSCRIPTIONAL_REGULATION_BY_TP53	1.95	0.0000	***0.0114***
C5 GO Molecular Function	GOMF_MANNOSIDASE_ACTIVITY	1.95	0.0019	***0.0114***
C2 WikiPathways pathway	WP_TOLLLIKE_RECEPTOR_SIGNALING_PATHWAY	1.95	0.0000	***0.0114***
C5 GO Biological Process	GOBP_PROTEIN_K11_LINKED_UBIQUITINATION	1.95	0.0000	***0.0115***
C5 GO Biological Process	GOBP_LEUKOCYTE_MEDIATED_CYTOTOXICITY	1.94	0.0000	***0.0116***
C2 Reactome pathway	REACTOME_NEGATIVE_REGULATION_OF_MAPK_PATHWAY	1.94	0.0000	***0.0116***
C5 GO Biological Process	GOBP_REGULATION_OF_B_CELL_DIFFERENTIATION	1.94	0.0019	***0.0116***
C5 GO Molecular Function	GOMF_UBIQUITIN_LIKE_PROTEIN_BINDING	1.94	0.0000	***0.0117***
C5 GO Biological Process	GOBP_PROTEIN_LOCALIZATION_TO_ENDOSOME	1.94	0.0000	***0.0118***
C5 GO Cellular Component	GOCC_MITOCHONDRIAL_PROTEIN_CONTAINING_COMPLEX	1.94	0.0000	***0.0119***
C2 Reactome pathway	REACTOME_MRNA_SPLICING	1.94	0.0000	***0.0119***
C2 Reactome pathway	REACTOME_RHOA_GTPASE_CYCLE	1.94	0.0000	***0.0119***
C5 GO Biological Process	GOBP_ALPHA_BETA_T_CELL_PROLIFERATION	1.94	0.0000	***0.0119***
C5 GO Biological Process	GOBP_TRANSLATIONAL_INITIATION	1.94	0.0000	***0.0120***
C2 Reactome pathway	REACTOME_COPII_MEDIATED_VESICLE_TRANSPORT	1.93	0.0000	***0.0121***
C2 Reactome pathway	REACTOME_RNA_POLYMERASE_II_TRANSCRIBES_SNRNA_GENES	1.93	0.0000	***0.0121***
C5 GO Biological Process	GOBP_ORGANELLE_DISASSEMBLY	1.93	0.0000	***0.0121***
C5 GO Biological Process	GOBP_CYTOSOLIC_TRANSPORT	1.94	0.0000	***0.0121***
C5 GO Biological Process	GOBP_MITOPHAGY	1.93	0.0018	***0.0121***
C2 KEGG pathway	KEGG_NEUROTROPHIN_SIGNALING_PATHWAY	1.93	0.0000	***0.0121***
C2 PID pathway	PID_IL6_7_PATHWAY	1.93	0.0000	***0.0121***
C5 GO Cellular Component	GOCC_INTRINSIC_COMPONENT_OF_ENDOPLASMIC_RETICULUM_MEMBRANE	1.94	0.0017	***0.0121***
C2 Reactome pathway	REACTOME_SWITCHING_OF_ORIGINS_TO_A_POST_REPLICATIVE_STATE	1.93	0.0000	***0.0121***
C5 GO Biological Process	GOBP_POSITIVE_REGULATION_OF_TELOMERASE_RNA_LOCALIZATION_TO_CAJAL_BODY	1.93	0.0038	***0.0121***
C2 WikiPathways pathway	WP_ATM_SIGNALING_PATHWAY	1.94	0.0017	***0.0121***

**Group**	**Gene Set Name**	**NES**	***p***	**FDR *q***

		post, trained vs control		
C2 WikiPathways pathway	WP_INTERFERON_TYPE_I_SIGNALING_PATHWAYS	1.93	0.0000	***0.0121***
C2 Reactome pathway	REACTOME_OVARIAN_TUMOR_DOMAIN_PROTEASES	1.93	0.0000	***0.0126***
C5 GO Biological Process	GOBP_INTRACELLULAR_PROTEIN_TRANSMEMBRANE_TRANSPORT	1.93	0.0036	***0.0126***
C2 Reactome pathway	REACTOME_SMAD2_SMAD3_SMAD4_HETEROTRIMER_REGULATES_TRANSCRIPTION	1.93	0.0000	***0.0126***
C2 PID pathway	PID_FCER1_PATHWAY	1.92	0.0000	***0.0128***
C2 PID pathway	PID_P53_REGULATION_PATHWAY	1.92	0.0000	***0.0128***
C2 Reactome pathway	REACTOME_TRANSCRIPTION_OF_THE_HIV_GENOME	1.92	0.0000	***0.0128***
C2 WikiPathways pathway	WP_ELECTRON_TRANSPORT_CHAIN_OXPHOS_SYSTEM_IN_MITOCHONDRIA	1.92	0.0000	***0.0128***
C2 Reactome pathway	REACTOME_CTLA4_INHIBITORY_SIGNALING	1.92	0.0000	***0.0129***
C5 GO Cellular Component	GOCC_AZUROPHIL_GRANULE_LUMEN	1.92	0.0000	***0.0129***
C5 GO Molecular Function	GOMF_MODIFICATION_DEPENDENT_PROTEIN_BINDING	1.92	0.0000	***0.0129***
C2 WikiPathways pathway	WP_MITOCHONDRIAL_IMMUNE_RESPONSE_TO_SARSCOV2	1.92	0.0000	***0.0129***
C2 Reactome pathway	REACTOME_PTEN_REGULATION	1.92	0.0000	***0.0129***
C5 GO Biological Process	GOBP_POSITIVE_REGULATION_OF_OXIDATIVE_STRESS_INDUCED_CELL_DEATH	1.92	0.0018	***0.0129***
C2 Reactome pathway	REACTOME_PARASITE_INFECTION	1.92	0.0000	***0.0129***
C2 PID pathway	PID_IL2_STAT5_PATHWAY	1.92	0.0019	***0.0129***
C2 PID pathway	PID_INSULIN_GLUCOSE_PATHWAY	1.92	0.0000	***0.0129***
C2 PID pathway	PID_IL2_1PATHWAY	1.92	0.0000	***0.0130***
C5 GO Biological Process	GOBP_REGULATION_OF_ALPHA_BETA_T_CELL_ACTIVATION	1.91	0.0000	***0.0130***
C5 GO Biological Process	GOBP_REGULATION_OF_ANTIGEN_RECEPTOR_MEDIATED_SIGNALING_PATHWAY	1.92	0.0000	***0.0130***
C5 GO Biological Process	GOBP_GOLGI_ORGANIZATION	1.91	0.0000	***0.0130***
C2 Reactome pathway	REACTOME_SIGNALING_BY_BRAF_AND_RAF1_FUSIONS	1.91	0.0000	***0.0130***
C2 Reactome pathway	REACTOME_REGULATION_OF_MRNA_STABILITY_BY_PROTEINS_THAT_BIND_AU_RICH_ELEMENTS	1.92	0.0000	***0.0130***
C2 Reactome pathway	REACTOME_CYTOSOLIC_SENSORS_OF_PATHOGEN_ASSOCIATED_DNA	1.92	0.0000	***0.0130***
C2 Reactome pathway	REACTOME_FCGAMMA_RECEPTOR_FCGR_DEPENDENT_PHAGOCYTOSIS	1.92	0.0000	***0.0131***
C5 GO Cellular Component	GOCC_INNER_MITOCHONDRIAL_MEMBRANE_PROTEIN_COMPLEX	1.91	0.0000	***0.0131***
C5 GO Cellular Component	GOCC_SPLICEOSOMAL_COMPLEX	1.91	0.0000	***0.0131***
C2 Reactome pathway	REACTOME_TOLL_LIKE_RECEPTOR_9_TLR9_CASCADE	1.92	0.0000	***0.0131***

C2 Reactome pathway	REACTOME_DEGRADATION_OF_BETA_CATENIN_BY_THE_DESTRUCTION_COMPLEX	1.91	0.0000	***0.0131***
C2 Reactome pathway	REACTOME_SIGNALING_BY_CTNNB1_PHOSPHO_SITE_MUTANTS	1.91	0.0019	***0.0131***
C2 WikiPathways pathway	WP_DNA_IRDAMAGE_AND_CELLULAR_RESPONSE_VIA_ATR	1.91	0.0000	***0.0131***
C2 Reactome pathway	REACTOME_CELL_CYCLE_CHECKPOINTS	1.91	0.0000	***0.0131***
C5 GO Biological Process	GOBP_AEROBIC_RESPIRATION	1.91	0.0000	***0.0131***
C5 GO Cellular Component	GOCC_U2_TYPE_SPLICEOSOMAL_COMPLEX	1.91	0.0000	***0.0131***
C2 KEGG pathway	KEGG_INSULIN_SIGNALING_PATHWAY	1.91	0.0000	***0.0132***
C2 Reactome pathway	REACTOME_ABERRANT_REGULATION_OF_MITOTIC_EXIT_IN_CANCER_DUE_TO_RB1_DEFECTS	1.91	0.0019	***0.0132***
C5 GO Cellular Component	GOCC_LUMENAL_SIDE_OF_MEMBRANE	1.91	0.0000	***0.0133***
C2 WikiPathways pathway	WP_TNFRELATED_WEAK_INDUCER_OF_APOPTOSIS_TWEAK_SIGNALING_PATHWAY	1.91	0.0000	***0.0135***
C5 GO Biological Process	GOBP_FATTY_ACID_BETA_OXIDATION	1.90	0.0000	***0.0140***
C2 Reactome pathway	REACTOME_PEROXISOMAL_PROTEIN_IMPORT	1.90	0.0000	***0.0141***
C2 Reactome pathway	REACTOME_RUNX1_REGULATES_GENES_INVOLVED_IN_MEGAKARYOCYTE_DIFFERENTIATION_AND_PLATELET_FUNCTION	1.90	0.0000	***0.0141***
C5 GO Biological Process	GOBP_NEGATIVE_REGULATION_OF_ENDOPLASMIC_RETICULUM_STRESS_INDUCED_INTRINSIC_APOPTOTIC_SIGNALING_PATHWAY	1.90	0.0000	***0.0142***

**Group**	**Gene Set Name**	**NES**	***p***	**FDR *q***
		**post, trained vs control**		
C5 GO Biological Process	GOBP_PROTEASOMAL_PROTEIN_CATABOLIC_PROCESS	1.90	0.0000	***0.0142***
C2 Reactome pathway	REACTOME_TOLL_LIKE_RECEPTOR_TLR1_TLR2_CASCADE	1.90	0.0000	***0.0143***
C2 Reactome pathway	REACTOME_DEFECTIVE_INTRINSIC_PATHWAY_FOR_APOPTOSIS	1.90	0.0000	***0.0143***
C5 GO Cellular Component	GOCC_SITE_OF_DOUBLE_STRAND_BREAK	1.90	0.0000	***0.0149***
C5 GO Biological Process	GOBP_PROTEIN_K63_LINKED_DEUBIQUITINATION	1.89	0.0017	***0.0149***
C2 KEGG pathway	KEGG_PANCREATIC_CANCER	1.89	0.0000	***0.0150***
C5 GO Biological Process	GOBP_POSITIVE_REGULATION_OF_ESTABLISHMENT_OF_PROTEIN_LOCALIZATION_TO_MITOCHONDRION	1.89	0.0019	***0.0150***
C5 GO Molecular Function	GOMF_RNA_POLYMERASE_CORE_ENZYME_BINDING	1.89	0.0000	***0.0150***
C2 Reactome pathway	REACTOME_REGULATION_OF_RUNX3_EXPRESSION_AND_ACTIVITY	1.89	0.0000	***0.0151***
C2 WikiPathways pathway	WP_CARDIAC_HYPERTROPHIC_RESPONSE	1.89	0.0017	***0.0151***
C5 GO Biological Process	GOBP_REGULATION_OF_HISTONE_METHYLATION	1.89	0.0000	***0.0151***

C2 Reactome pathway	REACTOME_ONCOGENIC_MAPK_SIGNALING	1.89	0.0000	***0.0151***
C2 Reactome pathway	REACTOME_REGULATION_OF_SIGNALING_BY_CBL	1.89	0.0000	***0.0152***
C2 Reactome pathway	REACTOME_RESPIRATORY_ELECTRON_TRANSPORT_ATP_SYNTHESIS_BY_CHEMIOSMOTIC_COUPLING_AND_HEAT_PRODUCTION_BY_UNCOUPLING_PROTEINS	1.89	0.0000	***0.0153***
C2 WikiPathways pathway	WP_PROTEASOME_DEGRADATION	1.89	0.0000	***0.0154***
C2 Reactome pathway	REACTOME_INTERLEUKIN_1_SIGNALING	1.89	0.0000	***0.0154***
C2 Reactome pathway	REACTOME_SYNTHESIS_OF_DNA	1.89	0.0000	***0.0155***
C2 WikiPathways pathway	WP_IL2_SIGNALING_PATHWAY	1.89	0.0000	***0.0155***
C5 GO Cellular Component	GOCC_MAGNESIUM_DEPENDENT_PROTEIN_SERINE_THREONINE_PHOSPHATASE_COMPLEX	1.89	0.0038	***0.0156***
C2 KEGG pathway	KEGG_ANTIGEN_PROCESSING_AND_PRESENTATION	1.89	0.0000	***0.0156***
C5 GO Biological Process	GOBP_VACUOLAR_TRANSPORT	1.89	0.0000	***0.0156***
C2 PID pathway	PID_CERAMIDE_PATHWAY	1.88	0.0000	***0.0158***
C5 GO Biological Process	GOBP_REGULATION_OF_LYMPHOCYTE_ACTIVATION	1.88	0.0000	***0.0158***
C5 GO Biological Process	GOBP_ESTABLISHMENT_OF_PROTEIN_LOCALIZATION_TO_ORGANELLE	1.88	0.0000	***0.0159***
C2 WikiPathways pathway	WP_NAD_METABOLISM_IN_ONCOGENEINDUCED_SENESCENCE_AND_MITOCHONDRIAL_DYSFUNCTIONASSOCIATED_SENESCENCE	1.88	0.0000	***0.0159***
C5 GO Biological Process	GOBP_B_CELL_ACTIVATION	1.88	0.0000	***0.0159***
C5 GO Molecular Function	GOMF_HEAT_SHOCK_PROTEIN_BINDING	1.88	0.0000	***0.0159***
C2 Reactome pathway	REACTOME_UB_SPECIFIC_PROCESSING_PROTEASES	1.88	0.0000	***0.0159***
C5 GO Cellular Component	GOCC_PHOSPHATASE_COMPLEX	1.88	0.0000	***0.0161***
C5 GO Molecular Function	GOMF_PHOSPHATASE_BINDING	1.88	0.0000	***0.0162***
C2 Reactome pathway	REACTOME_TELOMERE_EXTENSION_BY_TELOMERASE	1.88	0.0000	***0.0162***
C2 Reactome pathway	REACTOME_INTEGRIN_SIGNALING	1.88	0.0019	***0.0163***
C5 GO Molecular Function	GOMF_PROTEIN_PHOSPHORYLATED_AMINO_ACID_BINDING	1.88	0.0000	***0.0166***
C5 GO Cellular Component	GOCC_SITE_OF_DNA_DAMAGE	1.88	0.0000	***0.0166***
C5 GO Cellular Component	GOCC_VESICLE_LUMEN	1.88	0.0000	***0.0167***
C5 GO Biological Process	GOBP_POSITIVE_REGULATION_OF_HISTONE_MODIFICATION	1.87	0.0000	***0.0169***
C5 GO Cellular Component	GOCC_CHAPERONE_COMPLEX	1.87	0.0000	***0.0169***
C5 GO Cellular Component	GOCC_OUTER_MITOCHONDRIAL_MEMBRANE_PROTEIN_COMPLEX	1.87	0.0000	***0.0169***
C2 BioCarta pathway	BIOCARTA_BCR_PATHWAY	1.87	0.0000	***0.0169***
C2 Reactome pathway	REACTOME_HIV_LIFE_CYCLE	1.87	0.0000	***0.0169***

C5 GO Biological Process	GOBP_REGULATION_OF_PROTEASOMAL_PROTEIN_CATABOLIC_PROCESS	1.87	0.0000	***0.0169***
C2 Reactome pathway	REACTOME_DISEASES_OF_SIGNAL_TRANSDUCTION_BY_GROWTH_FACTOR_RECEPTORS_AND_SECOND_MESSENGERS	1.87	0.0000	***0.0169***

Group	Gene Set Name	NES	*p*	FDR *q*
		**post, trained vs control**		
C2 WikiPathways pathway	WP_COPPER_HOMEOSTASIS	1.87	0.0000	***0.0170***
C5 GO Molecular Function	GOMF_PHOSPHOLIPID_TRANSFER_ACTIVITY	1.87	0.0056	***0.0170***
C2 WikiPathways pathway	WP_TRANSLATION_INHIBITORS_IN_CHRONICALLY_ACTIVATED_PDGFRA_CELLS	1.87	0.0000	***0.0171***
C2 Reactome pathway	REACTOME_AUTOPHAGY	1.87	0.0000	***0.0174***
C5 GO Biological Process	GOBP_RNA_SPLICING_VIA_TRANSESTERIFICATION_REACTIONS	1.87	0.0000	***0.0174***
C2 Reactome pathway	REACTOME_UNFOLDED_PROTEIN_RESPONSE_UPR	1.87	0.0000	***0.0174***
C2 KEGG pathway	KEGG_T_CELL_RECEPTOR_SIGNALING_PATHWAY	1.87	0.0000	***0.0175***
C2 Reactome pathway	REACTOME_ASSOCIATION_OF_TRIC_CCT_WITH_TARGET_PROTEINS_DURING_BIOSYNTHESIS	1.87	0.0000	***0.0176***
C2 Reactome pathway	REACTOME_RRNA_MODIFICATION_IN_THE_NUCLEUS_AND_CYTOSOL	1.87	0.0000	***0.0176***
C2 Reactome pathway	REACTOME_DNA_REPLICATION	1.87	0.0000	***0.0176***
C2 Reactome pathway	REACTOME_INTERLEUKIN_1_FAMILY_SIGNALING	1.86	0.0000	***0.0177***
C2 Reactome pathway	REACTOME_UCH_PROTEINASES	1.86	0.0000	***0.0177***
C5 GO Biological Process	GOBP_CHAPERONE_MEDIATED_PROTEIN_FOLDING	1.86	0.0036	***0.0177***
C2 PID pathway	PID_VEGFR1_2_PATHWAY	1.86	0.0000	***0.0177***
C2 WikiPathways pathway	WP_DNA_IRDOUBLE_STRAND_BREAKS_AND_CELLULAR_RESPONSE_VIA_ATM	1.86	0.0035	***0.0178***
C2 WikiPathways pathway	WP_REGUCALCIN_IN_PROXIMAL_TUBULE_EPITHELIAL_KIDNEY_CELLS	1.86	0.0019	***0.0178***
C2 KEGG pathway	KEGG_PROTEIN_EXPORT	1.86	0.0000	***0.0179***
C2 WikiPathways pathway	WP_TCA_CYCLE_AKA_KREBS_OR_CITRIC_ACID_CYCLE	1.86	0.0018	***0.0179***
C2 Reactome pathway	REACTOME_THE_CITRIC_ACID_TCA_CYCLE_AND_RESPIRATORY_ELECTRON_TRANSPORT	1.86	0.0000	***0.0179***
C2 KEGG pathway	KEGG_PARKINSONS_DISEASE	1.86	0.0000	***0.0179***
C5 GO Biological Process	GOBP_RNA_SPLICING	1.86	0.0000	***0.0180***
C2 KEGG pathway	KEGG_UBIQUITIN_MEDIATED_PROTEOLYSIS	1.86	0.0000	***0.0180***
C5 GO Biological Process	GOBP_PROTEIN_TRANSMEMBRANE_TRANSPORT	1.86	0.0000	***0.0180***
C2 BioCarta pathway	BIOCARTA_NTHI_PATHWAY	1.86	0.0018	***0.0180***
C5 GO Molecular Function	GOMF_UNFOLDED_PROTEIN_BINDING	1.86	0.0000	***0.0182***

C2 Reactome pathway	REACTOME_PROTEIN_FOLDING	1.86	0.0017	***0.0182***
C5 GO Biological Process	GOBP_REGULATION_OF_LEUKOCYTE_DEGRANULATION	1.86	0.0037	***0.0183***
C5 GO Biological Process	GOBP_RESPIRATORY_ELECTRON_TRANSPORT_CHAIN	1.86	0.0000	***0.0183***
C5 GO Biological Process	GOBP_GRANULOCYTE_ACTIVATION	1.86	0.0000	***0.0184***
C5 GO Biological Process	GOBP_FC_EPSILON_RECEPTOR_SIGNALING_PATHWAY	1.86	0.0019	***0.0184***
C2 Reactome pathway	REACTOME_MAPK6_MAPK4_SIGNALING	1.86	0.0000	***0.0184***
C5 GO Cellular Component	GOCC_VESICLE_COAT	1.85	0.0000	***0.0185***
C5 GO Biological Process	GOBP_ANTIGEN_PROCESSING_AND_PRESENTATION	1.85	0.0000	***0.0189***
C5 GO Cellular Component	GOCC_PRESPLICEOSOME	1.85	0.0073	***0.0189***
C5 GO Biological Process	GOBP_FC_RECEPTOR_MEDIATED_STIMULATORY_SIGNALI NG_PATHWAY	1.85	0.0054	***0.0192***
C5 GO Cellular Component	GOCC_VACUOLAR_MEMBRANE	1.85	0.0000	***0.0193***
C5 GO Cellular Component	GOCC_SM_LIKE_PROTEIN_FAMILY_COMPLEX	1.85	0.0000	***0.0193***
C5 GO Biological Process	GOBP_ANTIGEN_PROCESSING_AND_PRESENTATION_OF_ PEPTIDE_ANTIGEN	1.85	0.0018	***0.0193***
C5 GO Biological Process	GOBP_TOLL_LIKE_RECEPTOR_SIGNALING_PATHWAY	1.85	0.0000	***0.0193***
C5 GO Biological Process	GOBP_REGULATION_OF_PROTEIN_DEPHOSPHORYLATION	1.85	0.0000	***0.0193***
C5 GO Cellular Component	GOCC_CUL4_RING_E3_UBIQUITIN_LIGASE_COMPLEX	1.85	0.0000	***0.0193***
C5 GO Biological Process	GOBP_CELLULAR_RESPONSE_TO_DSRNA	1.85	0.0000	***0.0193***

**Group**	**Gene Set Name**	**NES**	***p***	**FDR *q***
		**post, trained vs control**		
C2 Reactome pathway	REACTOME_ANTIGEN_PROCESSING_UBIQUITINATION_PR OTEASOME_DEGRADATION	1.84	0.0000	***0.0194***
C5 GO Molecular Function	GOMF_RNA_POLYMERASE_BINDING	1.85	0.0000	***0.0194***
C5 GO Biological Process	GOBP_B_CELL_DIFFERENTIATION	1.85	0.0000	***0.0194***
C2 WikiPathways pathway	WP_VEGFAVEGFR2_SIGNALING_PATHWAY	1.85	0.0000	***0.0194***
C2 Reactome pathway	REACTOME_THE_ROLE_OF_NEF_IN_HIV_1_REPLICATION _AND_DISEASE_PATHOGENESIS	1.85	0.0039	***0.0195***
C5 GO Biological Process	GOBP_VIRAL_RNA_GENOME_REPLICATION	1.85	0.0018	***0.0195***
C2 Reactome pathway	REACTOME_RAB_REGULATION_OF_TRAFFICKING	1.84	0.0000	***0.0197***
C5 GO Biological Process	GOBP_POSITIVE_REGULATION_OF_PROTEIN_DEPHOSPH ORYLATION	1.84	0.0018	***0.0197***
C5 GO Biological Process	GOBP_TRANSPORT_OF_VIRUS	1.84	0.0019	***0.0197***
C5 GO Biological Process	GOBP_RESPONSE_TO_LECTIN	1.84	0.0000	***0.0197***
C5 GO Biological Process	GOBP_PROTEIN_INSERTION_INTO_ER_MEMBRANE	1.84	0.0018	***0.0197***
C5 GO Biological Process	GOBP_ATP_METABOLIC_PROCESS	1.84	0.0000	***0.0197***
C2 WikiPathways pathway	WP_TLR4_SIGNALING_AND_TOLERANCE	1.84	0.0019	***0.0197***

C2 BioCarta pathway	BIOCARTA_ERAD_PATHWAY	1.84	0.0020	***0.0198***
C2 Reactome pathway	REACTOME_DECTIN_1_MEDIATED_NONCANONICAL_NF_KB_SIGNALING	1.84	0.0000	***0.0198***
C5 GO Biological Process	GOBP_REGULATION_OF_T_CELL_ACTIVATION	1.84	0.0000	***0.0198***
C2 WikiPathways pathway	WP_HOSTPATHOGEN_INTERACTION_OF_HUMAN_CORONAVIRUSES_INTERFERON_INDUCTION	1.84	0.0000	***0.0198***
C2 Reactome pathway	REACTOME_NUCLEOTIDE_BINDING_DOMAIN_LEUCINE_RICH_REPEAT_CONTAINING_RECEPTOR_NLR_SIGNALING_PATHWAYS	1.84	0.0000	***0.0198***
C5 GO Molecular Function	GOMF_POLYUBIQUITIN_MODIFICATION_DEPENDENT_PROTEIN_BINDING	1.84	0.0000	***0.0198***
C5 GO Cellular Component	GOCC_AZUROPHIL_GRANULE	1.84	0.0000	***0.0198***
C5 GO Biological Process	GOBP_REGULATION_OF_CELLULAR_RESPIRATION	1.84	0.0000	***0.0198***
C5 GO Molecular Function	GOMF_ADP_BINDING	1.84	0.0000	***0.0199***
C5 GO Biological Process	GOBP_NEGATIVE_REGULATION_OF_FIBROBLAST_PROLIFERATION	1.84	0.0056	***0.0199***
C5 GO Cellular Component	GOCC_SMALL_SUBUNIT_PROCESSOME	1.84	0.0018	***0.0199***
C2 Reactome pathway	REACTOME_REGULATION_OF_CHOLESTEROL_BIOSYNTHESIS_BY_SREBP_SREBF	1.84	0.0000	***0.0199***
C2 PID pathway	PID_FOXO_PATHWAY	1.84	0.0000	***0.0200***
C2 Reactome pathway	REACTOME_SEPARATION_OF_SISTER_CHROMATIDS	1.83	0.0000	***0.0200***
C2 Reactome pathway	REACTOME_APC_C_CDH1_MEDIATED_DEGRADATION_OF_CDC20_AND_OTHER_APC_C_CDH1_TARGETED_PROTEINS_IN_LATE_MITOSIS_EARLY_G1	1.84	0.0000	***0.0200***
C2 Reactome pathway	REACTOME_C_TYPE_LECTIN_RECEPTORS_CLRS	1.83	0.0000	***0.0200***
C5 GO Molecular Function	GOMF_PROTEIN_SELF_ASSOCIATION	1.84	0.0017	***0.0201***
C5 GO Molecular Function	GOMF_PROTEIN_PHOSPHATASE_BINDING	1.84	0.0000	***0.0201***
C5 GO Cellular Component	GOCC_PROTON_TRANSPORTING_ATP_SYNTHASE_COMPLEX	1.84	0.0019	***0.0201***
C2 Reactome pathway	REACTOME_RHO_GTPASES_ACTIVATE_WASPS_AND_WAVES	1.83	0.0020	***0.0201***
C5 GO Biological Process	GOBP_GOLGI_TO_ENDOSOME_TRANSPORT	1.83	0.0056	***0.0201***
C2 Reactome pathway	REACTOME_TP53_REGULATES_METABOLIC_GENES	1.83	0.0000	***0.0201***
C2 BioCarta pathway	BIOCARTA_RELA_PATHWAY	1.83	0.0037	***0.0203***
C5 GO Biological Process	GOBP_REGULATION_OF_PROGRAMMED_NECROTIC_CELL_DEATH	1.83	0.0018	***0.0204***
C5 GO Molecular Function	GOMF_UBIQUITIN_BINDING	1.83	0.0000	***0.0204***
C5 GO Biological Process	GOBP_PROTEIN_LOCALIZATION_TO_CHROMOSOME_TELOMERIC_REGION	1.83	0.0000	***0.0204***
C5 GO Cellular Component	GOCC_PROTEASOME_COMPLEX	1.83	0.0035	***0.0204***

C2 Reactome pathway	REACTOME_DNA_REPLICATION_PRE_INITIATION	1.83	0.0000	***0.0204***
C5 GO Molecular Function	GOMF_TRANSLATION_REGULATOR_ACTIVITY_NUCLEIC_ACID_BINDING	1.83	0.0000	***0.0204***

Group	Gene Set Name	NES	*p*	FDR *q*
		**post, trained vs control**		
C2 Reactome pathway	REACTOME_METABOLISM_OF_POLYAMINES	1.83	0.0018	***0.0204***
C2 Reactome pathway	REACTOME_ORC1_REMOVAL_FROM_CHROMATIN	1.83	0.0018	***0.0204***
C2 Reactome pathway	REACTOME_ANTIGEN_PRESENTATION_FOLDING_ASSEMBLY_AND_PEPTIDE_LOADING_OF_CLASS_I_MHC	1.83	0.0056	***0.0204***
C2 KEGG pathway	KEGG_TOLL_LIKE_RECEPTOR_SIGNALING_PATHWAY	1.83	0.0000	***0.0204***
C2 KEGG pathway	KEGG_CYTOSOLIC_DNA_SENSING_PATHWAY	1.83	0.0036	***0.0205***
C2 Reactome pathway	REACTOME_DEFECTIVE_CFTR_CAUSES_CYSTIC_FIBROSIS	1.83	0.0019	***0.0205***
C5 GO Molecular Function	GOMF_TRANSLATION_FACTOR_ACTIVITY_RNA_BINDING	1.83	0.0000	***0.0205***
C2 Reactome pathway	REACTOME_TP53_REGULATES_TRANSCRIPTION_OF_DNA_REPAIR_GENES	1.83	0.0018	***0.0206***
C5 GO Biological Process	GOBP_PROTEIN_K48_LINKED_UBIQUITINATION	1.83	0.0000	***0.0206***
C2 Reactome pathway	REACTOME_RUNX1_REGULATES_TRANSCRIPTION_OF_GENES_INVOLVED_IN_DIFFERENTIATION_OF_HSCS	1.83	0.0000	***0.0207***
C2 Reactome pathway	REACTOME_MTOR_SIGNALLING	1.83	0.0035	***0.0208***
C2 Reactome pathway	REACTOME_MITOTIC_G2_G2_M_PHASES	1.82	0.0000	***0.0209***
C2 Reactome pathway	REACTOME_CITRIC_ACID_CYCLE_TCA_CYCLE	1.82	0.0037	***0.0209***
C2 WikiPathways pathway	WP_COHESIN_COMPLEX_CORNELIA_DE_LANGE_SYNDROME	1.82	0.0018	***0.0212***
C2 Reactome pathway	REACTOME_NEGATIVE_REGULATION_OF_NOTCH4_SIGNALING	1.82	0.0000	***0.0212***
C2 Reactome pathway	REACTOME_STABILIZATION_OF_P53	1.82	0.0020	***0.0213***
C5 GO Biological Process	GOBP_ALPHA_BETA_T_CELL_ACTIVATION	1.82	0.0000	***0.0215***
C2 WikiPathways pathway	WP_PI3KAKTMTOR_SIGNALING_PATHWAY_AND_THERAPEUTIC_OPPORTUNITIES	1.82	0.0018	***0.0217***
C2 Reactome pathway	REACTOME_TGF_BETA_RECEPTOR_SIGNALING_ACTIVATES_SMADS	1.82	0.0037	***0.0220***
C2 Reactome pathway	REACTOME_M_PHASE	1.82	0.0000	***0.0220***
C5 GO Biological Process	GOBP_REGULATION_OF_PHAGOCYTOSIS	1.82	0.0000	***0.0220***
C2 PID pathway	PID_IL12_STAT4_PATHWAY	1.82	0.0017	***0.0222***
C2 WikiPathways pathway	WP_EUKARYOTIC_TRANSCRIPTION_INITIATION	1.82	0.0018	***0.0222***
C5 GO Biological Process	GOBP_SELECTIVE_AUTOPHAGY	1.82	0.0000	***0.0222***
C2 WikiPathways pathway	WP_ANDROGEN_RECEPTOR_SIGNALING_PATHWAY	1.82	0.0000	***0.0223***

C5 GO Biological Process	GOBP_PROTEIN_TARGETING_TO_PEROXISOME	1.81	0.0019	***0.0223***
C2 Reactome pathway	REACTOME_DEGRADATION_OF_DVL	1.81	0.0000	***0.0226***
C5 GO Biological Process	GOBP_POSITIVE_REGULATION_OF_CELL_ACTIVATION	1.81	0.0000	***0.0229***
C2 KEGG pathway	KEGG_LYSOSOME	1.81	0.0000	***0.0230***
C2 Reactome pathway	REACTOME_REGULATION_OF_HMOX1_EXPRESSION_AND_ACTIVITY	1.81	0.0017	***0.0230***
C5 GO Biological Process	GOBP_CELL_KILLING	1.81	0.0000	***0.0232***
C2 BioCarta pathway	BIOCARTA_CSK_PATHWAY	1.81	0.0000	***0.0235***
C5 GO Biological Process	GOBP_CELL_DEATH_IN_RESPONSE_TO_OXIDATIVE_STRESS	1.81	0.0000	***0.0235***
C5 GO Cellular Component	GOCC_RNA_POLYMERASE_COMPLEX	1.81	0.0017	***0.0236***
C5 GO Biological Process	GOBP_POSITIVE_REGULATION_OF_LEUKOCYTE_CELL_CELL_ADHESION	1.81	0.0000	***0.0236***
C2 Reactome pathway	REACTOME_SIGNALING_BY_INTERLEUKINS	1.81	0.0000	***0.0237***
C2 PID pathway	PID_TGFBR_PATHWAY	1.81	0.0000	***0.0239***
C2 WikiPathways pathway	WP_T_CELL_RECEPTOR_AND_COSTIMULATORY_SIGNALING	1.81	0.0056	***0.0241***
C2 Reactome pathway	REACTOME_RAF_INDEPENDENT_MAPK1_3_ACTIVATION	1.80	0.0038	***0.0242***
C5 GO Biological Process	GOBP_RRNA_TRANSCRIPTION	1.80	0.0000	***0.0242***
C5 GO Molecular Function	GOMF_MACROMOLECULE_TRANSMEMBRANE_TRANSPORTER_ACTIVITY	1.80	0.0037	***0.0243***
C2 PID pathway	PID_MYC_PATHWAY	1.80	0.0038	***0.0243***

**Group**	**Gene Set Name**	**NES**	***p***	**FDR *q***
		**post, trained vs control**		
C5 GO Biological Process	GOBP_CHROMATIN_DISASSEMBLY	1.80	0.0057	***0.0243***
C5 GO Biological Process	GOBP_NUCLEAR_EXPORT	1.80	0.0000	***0.0243***
C2 PID pathway	PID_NFAT_3PATHWAY	1.80	0.0000	***0.0244***
C2 PID pathway	PID_CD8_TCR_DOWNSTREAM_PATHWAY	1.80	0.0018	***0.0244***
C5 GO Molecular Function	GOMF_PEPTIDASE_ACTIVATOR_ACTIVITY	1.80	0.0000	***0.0244***
C2 Reactome pathway	REACTOME_GOLGI_TO_ER_RETROGRADE_TRANSPORT	1.80	0.0000	***0.0244***
C2 BioCarta pathway	BIOCARTA_TCR_PATHWAY	1.80	0.0035	***0.0245***
C2 Reactome pathway	REACTOME_CDT1_ASSOCIATION_WITH_THE_CDC6_ORC_ORIGIN_COMPLEX	1.80	0.0000	***0.0245***
C2 Reactome pathway	REACTOME_INHIBITION_OF_DNA_RECOMBINATION_AT_TELOMERE	1.80	0.0053	***0.0245***
C5 GO Biological Process	GOBP_NUCLEAR_TRANSPORT	1.80	0.0000	***0.0245***
C2 KEGG pathway	KEGG_CITRATE_CYCLE_TCA_CYCLE	1.80	0.0000	***0.0246***
C2 Reactome pathway	REACTOME_CELL_CYCLE_MITOTIC	1.80	0.0000	***0.0246***

C2 KEGG pathway	KEGG_OXIDATIVE_PHOSPHORYLATION	1.80	0.0000	***0.0246***
C2 Reactome pathway	REACTOME_DEGRADATION_OF_GLI1_BY_THE_PROTEASOME	1.80	0.0000	***0.0248***
C5 GO Cellular Component	GOCC_COPI_COATED_VESICLE	1.80	0.0037	***0.0249***
C5 GO Cellular Component	GOCC_TRANSCRIPTION_ELONGATION_FACTOR_COMPLEX	1.80	0.0018	***0.0251***
C2 Reactome pathway	REACTOME_MITOTIC_METAPHASE_AND_ANAPHASE	1.80	0.0000	***0.0251***
C5 GO Biological Process	GOBP_MAST_CELL_ACTIVATION_INVOLVED_IN_IMMUNE_RESPONSE	1.80	0.0000	***0.0252***
C2 WikiPathways pathway	WP_HEMATOPOIETIC_STEM_CELL_GENE_REGULATION_BY_GABP_ALPHABETA_COMPLEX	1.79	0.0000	***0.0252***
C2 WikiPathways pathway	WP_NETRINUNC5B_SIGNALING_PATHWAY	1.80	0.0018	***0.0252***
C5 GO Biological Process	GOBP_IMMUNOGLOBULIN_PRODUCTION	1.79	0.0000	***0.0252***
C5 GO Biological Process	GOBP_ATP_BIOSYNTHETIC_PROCESS	1.79	0.0000	***0.0252***
C2 WikiPathways pathway	WP_TYPE_II_DIABETES_MELLITUS	1.79	0.0055	***0.0252***
C5 GO Biological Process	GOBP_REGULATION_OF_TRANSLATION_IN_RESPONSE_TO_STRESS	1.79	0.0073	***0.0252***
C5 GO Biological Process	GOBP_SPLICEOSOMAL_COMPLEX_ASSEMBLY	1.79	0.0000	***0.0252***
C5 GO Biological Process	GOBP_ACTIVATION_OF_CYSTEINE_TYPE_ENDOPEPTIDASE_ACTIVITY_INVOLVED_IN_APOPTOTIC_PROCESS	1.79	0.0000	***0.0252***
C5 GO Biological Process	GOBP_CELLULAR_RESPONSE_TO_PROSTAGLANDIN_E_STIMULUS	1.79	0.0075	***0.0253***
C5 GO Biological Process	GOBP_FC_RECEPTOR_SIGNALING_PATHWAY	1.79	0.0034	***0.0253***
C2 Reactome pathway	REACTOME_INTERLEUKIN_17_SIGNALING	1.79	0.0000	***0.0253***
C2 Reactome pathway	REACTOME_RNA_POLYMERASE_III_TRANSCRIPTION_INITIATION_FROM_TYPE_1_PROMOTER	1.79	0.0000	***0.0253***
C5 GO Biological Process	GOBP_ENDOCRINE_HORMONE_SECRETION	-2.22	0.0000	***0.0253***
C5 GO Biological Process	GOBP_REGULATION_OF_CELLULAR_AMIDE_METABOLIC_PROCESS	1.79	0.0000	***0.0255***
C2 WikiPathways pathway	WP_ATM_SIGNALING_IN_DEVELOPMENT_AND_DISEASE	1.79	0.0000	***0.0257***
C5 GO Biological Process	GOBP_ATP_SYNTHESIS_COUPLED_ELECTRON_TRANSPORT	1.79	0.0000	***0.0258***
C5 GO Biological Process	GOBP_ENERGY_DERIVATION_BY_OXIDATION_OF_ORGANIC_COMPOUNDS	1.79	0.0000	***0.0259***
C2 Reactome pathway	REACTOME_CYCLIN_A_CDK2_ASSOCIATED_EVENTS_AT_S_PHASE_ENTRY	1.79	0.0000	***0.0260***
C5 GO Biological Process	GOBP_HISTONE_H4_K16_ACETYLATION	1.79	0.0053	***0.0262***
C5 GO Biological Process	GOBP_REGULATION_OF_PROTEIN_STABILITY	1.79	0.0000	***0.0262***
C5 GO Biological Process	GOBP_MRNA_PROCESSING	1.79	0.0000	***0.0262***
C5 GO Biological Process	GOBP_REGULATION_OF_HISTONE_MODIFICATION	1.79	0.0000	***0.0262***
C5 GO Biological Process	GOBP_LYSOSOMAL_TRANSPORT	1.79	0.0000	***0.0263***

C2 Reactome pathway	REACTOME_HIV_ELONGATION_ARREST_AND_RECOVERY	1.79	0.0019	***0.0263***

Group	Gene Set Name	NES	*p*	FDR *q*
		**post, trained vs control**		
C2 Reactome pathway	REACTOME_DEGRADATION_OF_AXIN	1.78	0.0000	***0.0263***
C2 WikiPathways pathway	WP_ESTROGEN_SIGNALING_PATHWAY	1.79	0.0055	***0.0264***
C2 Reactome pathway	REACTOME_INACTIVATION_OF_CSF3_G_CSF_SIGNALING	1.79	0.0037	***0.0264***
C2 Reactome pathway	REACTOME_FCERI_MEDIATED_CA_2_MOBILIZATION	1.78	0.0074	***0.0264***
C2 PID pathway	PID_MTOR_4PATHWAY	1.79	0.0017	***0.0264***
C2 KEGG pathway	KEGG_PEROXISOME	1.79	0.0000	***0.0264***
C5 GO Biological Process	GOBP_TRANSCRIPTION_BY_RNA_POLYMERASE_I	1.78	0.0035	***0.0264***
C5 GO Biological Process	GOBP_NEGATIVE_REGULATION_OF_ALPHA_BETA_T_CELL_ACTIVATION	1.78	0.0053	***0.0264***
Hallmark	HALLMARK_PEROXISOME	1.78	0.0000	***0.0264***
C5 GO Biological Process	GOBP_TELOMERASE_RNA_LOCALIZATION	1.78	0.0000	***0.0264***
C5 GO Biological Process	GOBP_POSITIVE_REGULATION_OF_HISTONE_METHYLATION	1.78	0.0000	***0.0265***
C5 GO Biological Process	GOBP_REGULATION_OF_GOLGI_ORGANIZATION	1.78	0.0037	***0.0265***
C2 Reactome pathway	REACTOME_THE_ROLE_OF_GTSE1_IN_G2_M_PROGRESSION_AFTER_G2_CHECKPOINT	1.78	0.0018	***0.0269***
C5 GO Biological Process	GOBP_NEGATIVE_REGULATION_OF_HISTONE_MODIFICATION	1.78	0.0000	***0.0271***
C5 GO Biological Process	GOBP_DNA_TEMPLATED_TRANSCRIPTION_INITIATION	1.78	0.0000	***0.0273***
C5 GO Biological Process	GOBP_GENERATION_OF_PRECURSOR_METABOLITES_AND_ENERGY	1.78	0.0000	***0.0274***
C5 GO Cellular Component	GOCC_RNA_POLYMERASE_II_HOLOENZYME	1.78	0.0000	***0.0276***
C5 GO Biological Process	GOBP_RNA_EXPORT_FROM_NUCLEUS	1.78	0.0019	***0.0277***
C2 BioCarta pathway	BIOCARTA_IL2RB_PATHWAY	1.78	0.0074	***0.0283***
C5 GO Biological Process	GOBP_POSITIVE_REGULATION_OF_LEUKOCYTE_DEGRANULATION	1.77	0.0114	***0.0284***
C2 Reactome pathway	REACTOME_RHOB_GTPASE_CYCLE	1.77	0.0000	***0.0284***
C5 GO Biological Process	GOBP_REGULATION_OF_REACTIVE_OXYGEN_SPECIES_BIOSYNTHETIC_PROCESS	1.77	0.0018	***0.0284***
C5 GO Biological Process	GOBP_MACROPHAGE_ACTIVATION_INVOLVED_IN_IMMUNE_RESPONSE	1.77	0.0057	***0.0284***
C5 GO Biological Process	GOBP_ELECTRON_TRANSPORT_CHAIN	1.78	0.0000	***0.0284***
C2 Reactome pathway	REACTOME_REGULATION_OF_TP53_ACTIVITY_THROUGH_METHYLATION	1.77	0.0036	***0.0284***
C5 GO Biological Process	GOBP_INTERMEMBRANE_LIPID_TRANSFER	1.77	0.0019	***0.0284***

C5 GO Biological Process	GOBP_LEUKOCYTE_CELL_CELL_ADHESION	1.77	0.0000	***0.0284***
C5 GO Molecular Function	GOMF_PROTEIN_TRANSMEMBRANE_TRANSPORTER_ACTIVITY	1.77	0.0037	***0.0284***
C2 Reactome pathway	REACTOME_SEALING_OF_THE_NUCLEAR_ENVELOPE_NE_BY_ESCRT_III	1.77	0.0155	***0.0285***
C5 GO Biological Process	GOBP_PATTERN_RECOGNITION_RECEPTOR_SIGNALING_PATHWAY	1.77	0.0000	***0.0285***
C2 Reactome pathway	REACTOME_REGULATION_OF_PTEN_STABILITY_AND_ACTIVITY	1.77	0.0018	***0.0285***
C2 Reactome pathway	REACTOME_ROLE_OF_PHOSPHOLIPIDS_IN_PHAGOCYTOSIS	1.77	0.0018	***0.0286***
C5 GO Biological Process	GOBP_PROTEIN_LOCALIZATION_TO_NUCLEUS	1.77	0.0000	***0.0286***
C2 Reactome pathway	REACTOME_CELLULAR_RESPONSE_TO_CHEMICAL_STRESS	1.77	0.0000	***0.0286***
C2 WikiPathways pathway	WP_EPO_RECEPTOR_SIGNALING	1.77	0.0036	***0.0287***
C2 WikiPathways pathway	WP_METABOLIC_REPROGRAMMING_IN_COLON_CANCER	1.77	0.0000	***0.0288***
C5 GO Biological Process	GOBP_NEGATIVE_REGULATION_OF_LYMPHOCYTE_ACTIVATION	1.77	0.0000	***0.0289***
C5 GO Biological Process	GOBP_VERY_LONG_CHAIN_FATTY_ACID_METABOLIC_PROCESS	1.77	0.0036	***0.0290***
C2 Reactome pathway	REACTOME_MITOCHONDRIAL_PROTEIN_IMPORT	1.77	0.0017	***0.0290***
C5 GO Molecular Function	GOMF_EXTRACELLULAR_MATRIX_STRUCTURAL_CONSTITUENT	-2.16	0.0000	***0.0290***
C2 WikiPathways pathway	WP_IL4_SIGNALING_PATHWAY	1.77	0.0054	***0.0291***
C5 GO Molecular Function	GOMF_BASAL_TRANSCRIPTION_MACHINERY_BINDING	1.77	0.0000	***0.0291***

**Group**	**Gene Set Name**	**NES**	***p***	**FDR *q***
		**post, trained vs control**		
C5 GO Biological Process	GOBP_NEGATIVE_REGULATION_OF_IMMUNE_EFFECTOR_PROCESS	1.77	0.0000	***0.0291***
C2 Reactome pathway	REACTOME_BETA_CATENIN_PHOSPHORYLATION_CASCADE	1.77	0.0057	***0.0292***
C5 GO Cellular Component	GOCC_PROTEASOME_CORE_COMPLEX	1.77	0.0000	***0.0292***
C2 Reactome pathway	REACTOME_CELLULAR_RESPONSE_TO_HYPOXIA	1.77	0.0000	***0.0292***
C2 Reactome pathway	REACTOME_TNFR2_NON_CANONICAL_NF_KB_PATHWAY	1.77	0.0017	***0.0292***
C2 PID pathway	PID_PI3KCI_AKT_PATHWAY	1.77	0.0018	***0.0294***
C2 KEGG pathway	KEGG_PROTEASOME	1.77	0.0019	***0.0295***
C2 Reactome pathway	REACTOME_CELLULAR_SENESCENCE	1.76	0.0000	***0.0296***
C5 GO Biological Process	GOBP_PROTEASOME_MEDIATED_UBIQUITIN_DEPENDENT_PROTEIN_CATABOLIC_PROCESS	1.76	0.0000	***0.0296***

C5 GO Biological Process	GOBP_REGULATION_OF_B_CELL_ACTIVATION	1.76	0.0017	***0.0298***
C5 GO Biological Process	GOBP_PROTEIN_MONOUBIQUITINATION	1.76	0.0017	***0.0300***
C2 WikiPathways pathway	WP_OXIDATIVE_PHOSPHORYLATION	1.76	0.0019	***0.0302***
C2 Reactome pathway	REACTOME_REGULATION_OF_TP53_ACTIVITY	1.76	0.0000	***0.0302***
C2 WikiPathways pathway	WP_CORI_CYCLE	1.76	0.0056	***0.0302***
C5 GO Biological Process	GOBP_POSITIVE_REGULATION_OF_TRANSLATION	1.76	0.0000	***0.0304***
C5 GO Molecular Function	GOMF_NUCLEAR_LOCALIZATION_SEQUENCE_BINDING	1.76	0.0078	***0.0304***
C5 GO Biological Process	GOBP_REGULATION_OF_B_CELL_PROLIFERATION	1.76	0.0000	***0.0304***
C2 BioCarta pathway	BIOCARTA_CERAMIDE_PATHWAY	1.76	0.0019	***0.0305***
C5 GO Biological Process	GOBP_LEUKOCYTE_PROLIFERATION	1.76	0.0000	***0.0305***
C5 GO Biological Process	GOBP_RIBONUCLEOSIDE_TRIPHOSPHATE_METABOLIC_PROCESS	1.76	0.0000	***0.0305***
C5 GO Biological Process	GOBP_ATP_SYNTHESIS_COUPLED_PROTON_TRANSPORT	1.76	0.0055	***0.0305***
C5 GO Molecular Function	GOMF_ELECTRON_TRANSFER_ACTIVITY	1.76	0.0000	***0.0305***
C2 Reactome pathway	REACTOME_DAP12_SIGNALING	1.76	0.0018	***0.0305***
C2 PID pathway	PID_PS1_PATHWAY	1.76	0.0035	***0.0305***
C5 GO Biological Process	GOBP_FIBROBLAST_APOPTOTIC_PROCESS	1.76	0.0019	***0.0305***
C5 GO Biological Process	GOBP_REGULATION_OF_IMMUNOGLOBULIN_PRODUCTION	1.76	0.0000	***0.0306***
C5 GO Biological Process	GOBP_CELLULAR_LIPID_CATABOLIC_PROCESS	1.76	0.0000	***0.0307***
C5 GO Cellular Component	GOCC_TRAPP_COMPLEX	1.76	0.0073	***0.0307***
C2 Reactome pathway	REACTOME_CYTOPROTECTION_BY_HMOX1	1.76	0.0000	***0.0307***
C2 Reactome pathway	REACTOME_APC_C_MEDIATED_DEGRADATION_OF_CELL_CYCLE_PROTEINS	1.76	0.0000	***0.0307***
C5 GO Biological Process	GOBP_POSITIVE_REGULATION_OF_PROTEASOMAL_UBIQUITIN_DEPENDENT_PROTEIN_CATABOLIC_PROCESS	1.76	0.0017	***0.0308***
C5 GO Biological Process	GOBP_ORGANELLE_INHERITANCE	1.75	0.0059	***0.0310***
C5 GO Biological Process	GOBP_ESTABLISHMENT_OF_PROTEIN_LOCALIZATION_TO_ENDOPLASMIC_RETICULUM	1.75	0.0052	***0.0311***
C2 Reactome pathway	REACTOME_TRANSCRIPTIONAL_REGULATION_BY_VENTX	1.75	0.0068	***0.0311***
C5 GO Biological Process	GOBP_CERAMIDE_CATABOLIC_PROCESS	1.75	0.0036	***0.0311***
C5 GO Biological Process	GOBP_POSITIVE_REGULATION_OF_ALPHA_BETA_T_CELL_ACTIVATION	1.75	0.0053	***0.0312***
C5 GO Biological Process	GOBP_RECEPTOR_CATABOLIC_PROCESS	1.75	0.0000	***0.0312***
C5 GO Biological Process	GOBP_NEGATIVE_REGULATION_OF_KINASE_ACTIVITY	1.75	0.0000	***0.0312***
C5 GO Biological Process	GOBP_NEGATIVE_REGULATION_OF_PEPTIDYL_LYSINE_ACETYLATION	1.75	0.0073	***0.0312***
C2 WikiPathways pathway	WP_IL7_SIGNALING_PATHWAY	1.75	0.0074	***0.0312***
C5 GO Biological Process	GOBP_MITOCHONDRIAL_TRANSMEMBRANE_TRANSPORT	1.75	0.0000	***0.0312***

C2 KEGG pathway	KEGG_ALLOGRAFT_REJECTION	1.75	0.0018	***0.0313***

**Group**	**Gene Set Name**	**NES**	***p***	**FDR *q***
		**post, trained vs control**		
C5 GO Cellular Component	GOCC_PHOTORECEPTOR_INNER_SEGMENT	-2.13	0.0000	***0.0315***
C2 Reactome pathway	REACTOME_ABC_FAMILY_PROTEINS_MEDIATED_TRANSPORT	1.75	0.0000	***0.0317***
C2 Reactome pathway	REACTOME_DUAL_INCISION_IN_TC_NER	1.75	0.0018	***0.0318***
C2 PID pathway	PID_IFNG_PATHWAY	1.75	0.0056	***0.0318***
C2 Reactome pathway	REACTOME_MAP2K_AND_MAPK_ACTIVATION	1.75	0.0018	***0.0318***
C5 GO Cellular Component	GOCC_COPI_COATED_VESICLE_MEMBRANE	1.75	0.0056	***0.0321***
C2 Reactome pathway	REACTOME_INTERLEUKIN_RECEPTOR_SHC_SIGNALING	1.75	0.0073	***0.0321***
C2 PID pathway	PID_MET_PATHWAY	1.75	0.0018	***0.0322***
C2 Reactome pathway	REACTOME_FATTY_ACYL_COA_BIOSYNTHESIS	1.75	0.0036	***0.0322***
C2 Reactome pathway	REACTOME_RAF_ACTIVATION	1.75	0.0019	***0.0324***
C2 PID pathway	PID_ECADHERIN_NASCENT_AJ_PATHWAY	1.75	0.0018	***0.0324***
C2 Reactome pathway	REACTOME_RHO_GTPASES_ACTIVATE_NADPH_OXIDASES	1.74	0.0058	***0.0327***
C2 Reactome pathway	REACTOME_PURINERGIC_SIGNALING_IN_LEISHMANIASIS_INFECTION	1.74	0.0091	***0.0327***
C5 GO Biological Process	GOBP_RNA_LOCALIZATION	1.74	0.0000	***0.0328***
C5 GO Biological Process	GOBP_LATE_ENDOSOME_TO_LYSOSOME_TRANSPORT	1.74	0.0038	***0.0328***
C5 GO Biological Process	GOBP_REGULATION_OF_SUPEROXIDE_ANION_GENERATION	1.74	0.0133	***0.0328***
C2 Reactome pathway	REACTOME_PROGRAMMED_CELL_DEATH	1.74	0.0000	***0.0328***
C2 BioCarta pathway	BIOCARTA_RHO_PATHWAY	1.74	0.0072	***0.0328***
C2 BioCarta pathway	BIOCARTA_MTOR_PATHWAY	1.74	0.0076	***0.0328***
C5 GO Biological Process	GOBP_POSITIVE_REGULATION_OF_DEPHOSPHORYLATION	1.74	0.0000	***0.0328***
C5 GO Biological Process	GOBP_POSITIVE_REGULATION_OF_IMMUNE_RESPONSE	1.74	0.0000	***0.0328***
C5 GO Molecular Function	GOMF_PHOSPHOTYROSINE_RESIDUE_BINDING	1.74	0.0034	***0.0328***
C5 GO Biological Process	GOBP_MONOCARBOXYLIC_ACID_CATABOLIC_PROCESS	1.74	0.0000	***0.0329***
C5 GO Biological Process	GOBP_PROTEIN_FOLDING	1.74	0.0000	***0.0329***
C2 Reactome pathway	REACTOME_TOLL_LIKE_RECEPTOR_CASCADES	1.74	0.0000	***0.0329***
C5 GO Biological Process	GOBP_REGULATION_OF_CELLULAR_PROTEIN_CATABOLIC_PROCESS	1.74	0.0000	***0.0329***
C5 GO Molecular Function	GOMF_LIPID_TRANSFER_ACTIVITY	1.74	0.0018	***0.0329***
C2 KEGG pathway	KEGG_PRIMARY_IMMUNODEFICIENCY	1.74	0.0018	***0.0329***
C5 GO Biological Process	GOBP_PROTEIN_IMPORT	1.74	0.0000	***0.0330***

C5 GO Biological Process	GOBP_NEGATIVE_REGULATION_OF_DNA_REPAIR	1.74	0.0072	***0.0331***
C5 GO Biological Process	GOBP_PEROXISOMAL_TRANSPORT	1.74	0.0074	***0.0331***
C2 PID pathway	PID_ERBB1_DOWNSTREAM_PATHWAY	1.74	0.0000	***0.0332***
C5 GO Biological Process	GOBP_RESPONSE_TO_ENDOPLASMIC_RETICULUM_STRESS	1.74	0.0000	***0.0332***
C2 WikiPathways pathway	WP_EBSTEINBARR_VIRUS_LMP1_SIGNALING	1.74	0.0104	***0.0332***
C5 GO Molecular Function	GOMF_MITOGEN_ACTIVATED_PROTEIN_KINASE_BINDING	1.74	0.0078	***0.0333***
C5 GO Biological Process	GOBP_DOUBLE_STRAND_BREAK_REPAIR_VIA_NONHOMOLOGOUS_END_JOINING	1.74	0.0017	***0.0334***
C2 BioCarta pathway	BIOCARTA_HIVNEF_PATHWAY	1.74	0.0000	***0.0334***
C2 PID pathway	PID_IGF1_PATHWAY	1.74	0.0093	***0.0337***
C5 GO Biological Process	GOBP_REGULATION_OF_PATTERN_RECOGNITION_RECEPTOR_SIGNALING_PATHWAY	1.74	0.0000	***0.0339***
C5 GO Molecular Function	GOMF_NON_MEMBRANE_SPANNING_PROTEIN_TYROSINE_KINASE_ACTIVITY	1.74	0.0017	***0.0339***
C2 KEGG pathway	KEGG_HUNTINGTONS_DISEASE	1.74	0.0000	***0.0341***
C5 GO Biological Process	GOBP_REGULATION_OF_RESPONSE_TO_DNA_DAMAGE_STIMULUS	1.74	0.0000	***0.0341***

Group	Gene Set Name	NES	*p*	FDR *q*
		post, trained vs control		
C5 GO Biological Process	GOBP_PROTEIN_TRANSMEMBRANE_IMPORT_INTO_INTRACELLULAR_ORGANELLE	1.74	0.0054	***0.0341***
C5 GO Biological Process	GOBP_RIBONUCLEOSIDE_TRIPHOSPHATE_BIOSYNTHETIC_PROCESS	1.73	0.0036	***0.0342***
C2 PID pathway	PID_AR_NONGENOMIC_PATHWAY	1.73	0.0019	***0.0342***
C2 WikiPathways pathway	WP_WNT_SIGNALING_PATHWAY	1.73	0.0072	***0.0346***
C5 GO Biological Process	GOBP_CARDIOLIPIN_METABOLIC_PROCESS	1.73	0.0150	***0.0347***
C5 GO Molecular Function	GOMF_UBIQUITIN_LIKE_PROTEIN_LIGASE_BINDING	1.73	0.0000	***0.0348***
C5 GO Biological Process	GOBP_POSITIVE_REGULATION_OF_INTERLEUKIN_2_PRODUCTION	1.73	0.0091	***0.0348***
C2 BioCarta pathway	BIOCARTA_TOLL_PATHWAY	1.73	0.0074	***0.0348***
C2 Reactome pathway	REACTOME_RAB_GEFS_EXCHANGE_GTP_FOR_GDP_ON_RABS	1.73	0.0000	***0.0348***
C5 GO Biological Process	GOBP_TRANSCRIPTION_BY_RNA_POLYMERASE_III	1.73	0.0036	***0.0348***
C2 Reactome pathway	REACTOME_CLASS_I_PEROXISOMAL_MEMBRANE_PROTEIN_IMPORT	1.73	0.0093	***0.0348***
C2 KEGG pathway	KEGG_INOSITOL_PHOSPHATE_METABOLISM	1.73	0.0035	***0.0349***

C5 GO Cellular Component	GOCC_PROTON_TRANSPORTING_TWO_SECTOR_ATPASE_COMPLEX	1.73	0.0018	***0.0349***
C5 GO Cellular Component	GOCC_PERICENTRIOLAR_MATERIAL	1.73	0.0074	***0.0349***
C5 GO Biological Process	GOBP_CELLULAR_RESPONSE_TO_EXOGENOUS_DSRNA	1.73	0.0039	***0.0349***
C2 Reactome pathway	REACTOME_MITOCHONDRIAL_BIOGENESIS	1.73	0.0018	***0.0352***
C5 GO Biological Process	GOBP_POSITIVE_REGULATION_OF_ANTIGEN_RECEPTOR_MEDIATED_SIGNALING_PATHWAY	1.73	0.0053	***0.0353***
C5 GO Cellular Component	GOCC_U2_SNRNP	1.73	0.0036	***0.0353***
C2 WikiPathways pathway	WP_MEASLES_VIRUS_INFECTION	1.73	0.0000	***0.0353***
C5 GO Biological Process	GOBP_RRNA_METABOLIC_PROCESS	1.73	0.0000	***0.0353***
C2 Reactome pathway	REACTOME_PHOSPHORYLATION_OF_THE_APC_C	1.73	0.0054	***0.0354***
C2 PID pathway	PID_CXCR4_PATHWAY	1.73	0.0017	***0.0354***
C2 WikiPathways pathway	WP_CELL_CYCLE	1.73	0.0000	***0.0354***
C2 Reactome pathway	REACTOME_TELOMERE_MAINTENANCE	1.73	0.0017	***0.0354***
C2 Reactome pathway	REACTOME_SIGNALING_BY_NOTCH	1.73	0.0000	***0.0354***
C2 WikiPathways pathway	WP_TRANSLATION_FACTORS	1.73	0.0036	***0.0355***
C5 GO Biological Process	GOBP_ENDOSOMAL_TRANSPORT	1.73	0.0000	***0.0355***
C2 KEGG pathway	KEGG_NON_SMALL_CELL_LUNG_CANCER	1.73	0.0017	***0.0355***
C5 GO Biological Process	GOBP_POSITIVE_REGULATION_OF_CELLULAR_PROTEIN_CATABOLIC_PROCESS	1.73	0.0000	***0.0355***
C5 GO Molecular Function	GOMF_UBIQUITIN_LIKE_PROTEIN_SPECIFIC_PROTEASE_ACTIVITY	1.72	0.0000	***0.0362***
C2 Reactome pathway	REACTOME_ACTIVATION_OF_GENE_EXPRESSION_BY_SREBF_SREBP	1.72	0.0018	***0.0362***
C2 BioCarta pathway	BIOCARTA_GH_PATHWAY	1.72	0.0074	***0.0362***
C2 Reactome pathway	REACTOME_AUF1_HNRNP_D0_BINDS_AND_DESTABILIZES_MRNA	1.72	0.0020	***0.0363***
C5 GO Biological Process	GOBP_NEGATIVE_REGULATION_OF_TRANSFERASE_ACTIVITY	1.72	0.0000	***0.0363***
C2 Reactome pathway	REACTOME_GLYCOSPHINGOLIPID_METABOLISM	1.72	0.0089	***0.0363***
C5 GO Biological Process	GOBP_CELL_ACTIVATION_INVOLVED_IN_IMMUNE_RESPONSE	1.72	0.0000	***0.0363***
C5 GO Biological Process	GOBP_MYELOID_LEUKOCYTE_MEDIATED_IMMUNITY	1.72	0.0034	***0.0364***
C5 GO Biological Process	GOBP_ACTIVATION_OF_INNATE_IMMUNE_RESPONSE	1.72	0.0000	***0.0364***
C2 Reactome pathway	REACTOME_PLATELET_ACTIVATION_SIGNALING_AND_AGGREGATION	1.72	0.0000	***0.0364***
C5 GO Biological Process	GOBP_REGULATION_OF_HISTONE_H3_K9_METHYLATION	1.72	0.0129	***0.0364***
C5 GO Biological Process	GOBP_AUTOPHAGY_OF_MITOCHONDRION	1.72	0.0000	***0.0365***
C5 GO Biological Process	GOBP_PROTEIN_POLYUBIQUITINATION	1.72	0.0000	***0.0365***

Group	Gene Set Name	NES	*p*	FDR *q*
		post, trained vs control		
C5 GO Biological Process	GOBP_MITOTIC_INTRA_S_DNA_DAMAGE_CHECKPOINT_SIGNALING	1.72	0.0060	***0.0366***
C5 GO Biological Process	GOBP_DNA_DOUBLE_STRAND_BREAK_PROCESSING	1.72	0.0055	***0.0368***
C2 Reactome pathway	REACTOME_CD28_CO_STIMULATION	1.72	0.0125	***0.0368***
C5 GO Biological Process	GOBP_COPII_COATED_VESICLE_CARGO_LOADING	1.72	0.0113	***0.0368***
C5 GO Biological Process	GOBP_NUCLEOBASE_BIOSYNTHETIC_PROCESS	1.72	0.0096	***0.0369***
C2 PID pathway	PID_ATR_PATHWAY	1.72	0.0075	***0.0369***
C2 Reactome pathway	REACTOME_REGULATION_OF_TP53_ACTIVITY_THROUGH_PHOSPHORYLATION	1.72	0.0017	***0.0369***
C5 GO Biological Process	GOBP_PROTEIN_QUALITY_CONTROL_FOR_MISFOLDED_OR_INCOMPLETELY_SYNTHESIZED_PROTEINS	1.72	0.0055	***0.0370***
C5 GO Biological Process	GOBP_REGULATION_OF_PROTEIN_CATABOLIC_PROCESS	1.72	0.0000	***0.0370***
C5 GO Molecular Function	GOMF_S_ACYLTRANSFERASE_ACTIVITY	1.72	0.0093	***0.0370***
C5 GO Biological Process	GOBP_CELLULAR_RESPONSE_TO_BIOTIC_STIMULUS	1.72	0.0000	***0.0370***
C5 GO Biological Process	GOBP_TUMOR_NECROSIS_FACTOR_MEDIATED_SIGNALING_PATHWAY	1.72	0.0017	***0.0370***
C5 GO Biological Process	GOBP_LEUKOCYTE_MEDIATED_IMMUNITY	1.72	0.0000	***0.0370***
C5 GO Biological Process	GOBP_ERAD_PATHWAY	1.72	0.0017	***0.0371***
C2 PID pathway	PID_TXA2PATHWAY	1.72	0.0018	***0.0371***
C2 Reactome pathway	REACTOME_RHOC_GTPASE_CYCLE	1.72	0.0000	***0.0371***
Hallmark	HALLMARK_REACTIVE_OXYGEN_SPECIES_PATHWAY	1.72	0.0018	***0.0371***
C2 Reactome pathway	REACTOME_PROCESSING_OF_DNA_DOUBLE_STRAND_BREAK_ENDS	1.72	0.0017	***0.0371***
C2 WikiPathways pathway	WP_APOPTOSIS	1.72	0.0000	***0.0371***
C2 KEGG pathway	KEGG_VASOPRESSIN_REGULATED_WATER_REABSORPTION	1.71	0.0037	***0.0373***
C5 GO Biological Process	GOBP_RNA_POLYADENYLATION	1.71	0.0035	***0.0373***
C2 BioCarta pathway	BIOCARTA_LONGEVITY_PATHWAY	1.71	0.0073	***0.0374***
C2 KEGG pathway	KEGG_NOTCH_SIGNALING_PATHWAY	1.71	0.0036	***0.0375***
C2 Reactome pathway	REACTOME_G1_S_DNA_DAMAGE_CHECKPOINTS	1.71	0.0052	***0.0376***
C5 GO Biological Process	GOBP_FC_GAMMA_RECEPTOR_SIGNALING_PATHWAY	1.71	0.0037	***0.0376***
C5 GO Biological Process	GOBP_POSITIVE_REGULATION_OF_TRANSLATIONAL_INITIATION	1.71	0.0072	***0.0377***
C5 GO Biological Process	GOBP_REGULATION_OF_HISTONE_H3_K4_METHYLATION	1.71	0.0037	***0.0378***
C5 GO Molecular Function	GOMF_PROMOTER_SPECIFIC_CHROMATIN_BINDING	1.71	0.0017	***0.0378***
C2 Reactome pathway	REACTOME_CD28_DEPENDENT_PI3K_AKT_SIGNALING	1.71	0.0098	***0.0378***

C5 GO Biological Process	GOBP_NEGATIVE_REGULATION_OF_PRODUCTION_OF_MOLECULAR_MEDIATOR_OF_IMMUNE_RESPONSE	1.71	0.0018	***0.0378***
C5 GO Biological Process	GOBP_FATTY_ACID_CATABOLIC_PROCESS	1.71	0.0000	***0.0379***
C5 GO Cellular Component	GOCC_SPECIFIC_GRANULE_LUMEN	1.71	0.0073	***0.0379***
C5 GO Biological Process	GOBP_REGULATION_OF_DEPHOSPHORYLATION	1.71	0.0000	***0.0380***
C2 Reactome pathway	REACTOME_TRANSPORT_OF_MATURE_TRANSCRIPT_TO_CYTOPLASM	1.71	0.0017	***0.0381***
C5 GO Cellular Component	GOCC_UBIQUITIN_LIGASE_COMPLEX	1.71	0.0000	***0.0382***
C2 Reactome pathway	REACTOME_DNA_DOUBLE_STRAND_BREAK_RESPONSE	1.71	0.0056	***0.0382***
C5 GO Biological Process	GOBP_REGULATION_OF_T_CELL_RECEPTOR_SIGNALING_PATHWAY	1.71	0.0000	***0.0385***
C5 GO Molecular Function	GOMF_PEPTIDASE_ACTIVATOR_ACTIVITY_INVOLVED_IN_APOPTOTIC_PROCESS	1.71	0.0038	***0.0385***
C2 Reactome pathway	REACTOME_REGULATION_OF_RAS_BY_GAPS	1.71	0.0018	***0.0385***
C5 GO Molecular Function	GOMF_ENZYME_ACTIVATOR_ACTIVITY	1.71	0.0000	***0.0386***
C5 GO Molecular Function	GOMF_OXIDOREDUCTASE_ACTIVITY_ACTING_ON_THE_CH_CH_GROUP_OF_DONORS	1.71	0.0109	***0.0387***
C2 Reactome pathway	REACTOME_GPVI_MEDIATED_ACTIVATION_CASCADE	1.71	0.0145	***0.0387***

**Group**	**Gene Set Name**	**NES**	***p***	**FDR *q***
		**post, trained vs control**		
C5 GO Biological Process	GOBP_REGULATION_OF_MYELOID_LEUKOCYTE_MEDIATED_IMMUNITY	1.71	0.0000	***0.0388***
C2 Reactome pathway	REACTOME_FOXO_MEDIATED_TRANSCRIPTION	1.71	0.0018	***0.0388***
C2 Reactome pathway	REACTOME_IRE1ALPHA_ACTIVATES_CHAPERONES	1.71	0.0000	***0.0388***
C2 BioCarta pathway	BIOCARTA_KERATINOCYTE_PATHWAY	1.70	0.0055	***0.0390***
C5 GO Biological Process	GOBP_HISTONE_UBIQUITINATION	1.70	0.0035	***0.0391***
C2 WikiPathways pathway	WP_KIT_RECEPTOR_SIGNALING_PATHWAY	1.70	0.0000	***0.0391***
C2 Reactome pathway	REACTOME_RETROGRADE_TRANSPORT_AT_THE_TRANS_GOLGI_NETWORK	1.70	0.0090	***0.0391***
C5 GO Biological Process	GOBP_VACUOLE_ORGANIZATION	1.70	0.0017	***0.0391***
C5 GO Biological Process	GOBP_REGULATION_OF_NUCLEASE_ACTIVITY	1.70	0.0092	***0.0391***
C2 Reactome pathway	REACTOME_RRNA_PROCESSING	1.70	0.0000	***0.0391***
C2 WikiPathways pathway	WP_VASOPRESSINREGULATED_WATER_REABSORPTION	1.70	0.0056	***0.0392***
C5 GO Biological Process	GOBP_ESTABLISHMENT_OF_PROTEIN_LOCALIZATION_TO_TELOMERE	1.70	0.0097	***0.0393***
C2 KEGG pathway	KEGG_PHOSPHATIDYLINOSITOL_SIGNALING_SYSTEM	1.70	0.0017	***0.0394***
C2 Reactome pathway	REACTOME_DAP12_INTERACTIONS	1.70	0.0058	***0.0394***

C5 GO Biological Process	GOBP_POSITIVE_REGULATION_OF_PROTEOLYSIS_INVOLVED_IN_CELLULAR_PROTEIN_CATABOLIC_PROCESS	1.70	0.0000	***0.0394***
C5 GO Cellular Component	GOCC_ENDOLYSOSOME	1.70	0.0055	***0.0394***
C5 GO Biological Process	GOBP_TAIL_ANCHORED_MEMBRANE_PROTEIN_INSERTION_INTO_ER_MEMBRANE	1.70	0.0075	***0.0395***
Hallmark	HALLMARK_UNFOLDED_PROTEIN_RESPONSE	1.70	0.0017	***0.0395***
C2 Reactome pathway	REACTOME_DEADENYLATION_DEPENDENT_MRNA_DECAY	1.70	0.0018	***0.0396***
C2 KEGG pathway	KEGG_ENDOMETRIAL_CANCER	1.70	0.0092	***0.0396***
C2 PID pathway	PID_EPO_PATHWAY	1.70	0.0037	***0.0397***
C5 GO Biological Process	GOBP_POSITIVE_REGULATION_OF_PROTEIN_LOCALIZATION_TO_NUCLEUS	1.70	0.0000	***0.0400***
C5 GO Biological Process	GOBP_T_CELL_PROLIFERATION	1.70	0.0000	***0.0400***
C5 GO Biological Process	GOBP_REGULATION_OF_REACTIVE_OXYGEN_SPECIES_METABOLIC_PROCESS	1.70	0.0000	***0.0401***
C2 PID pathway	PID_NCADHERIN_PATHWAY	1.70	0.0055	***0.0401***
C2 BioCarta pathway	BIOCARTA_IL7_PATHWAY	1.70	0.0078	***0.0401***
C2 PID pathway	PID_SMAD2_3NUCLEAR_PATHWAY	1.70	0.0017	***0.0403***
C2 Reactome pathway	REACTOME_HIV_TRANSCRIPTION_INITIATION	1.70	0.0051	***0.0405***
C2 Reactome pathway	REACTOME_NONHOMOLOGOUS_END_JOINING_NHEJ	1.70	0.0057	***0.0405***
C5 GO Molecular Function	GOMF_GTPASE_BINDING	1.70	0.0000	***0.0405***
C5 GO Cellular Component	GOCC_ORGANELLE_INNER_MEMBRANE	1.70	0.0000	***0.0409***
C5 GO Biological Process	GOBP_NCRNA_TRANSCRIPTION	1.69	0.0051	***0.0412***
C5 GO Biological Process	GOBP_TRANSLATIONAL_ELONGATION	1.69	0.0056	***0.0412***
C2 Reactome pathway	REACTOME_SIGNALING_BY_CSF3_G_CSF	1.69	0.0071	***0.0412***
C5 GO Biological Process	GOBP_POSITIVE_REGULATION_OF_PROTEIN_CATABOLIC_PROCESS	1.69	0.0000	***0.0416***
C5 GO Biological Process	GOBP_MATURATION_OF_LSU_RRNA	1.69	0.0130	***0.0416***
C5 GO Biological Process	GOBP_NEGATIVE_REGULATION_OF_PEPTIDYL_TYROSINE_PHOSPHORYLATION	1.69	0.0037	***0.0417***
C2 BioCarta pathway	BIOCARTA_TGFB_PATHWAY	1.69	0.0055	***0.0417***
C5 GO Cellular Component	GOCC_MEMBRANE_COAT	1.69	0.0036	***0.0417***
C2 BioCarta pathway	BIOCARTA_TNFR1_PATHWAY	1.69	0.0056	***0.0417***
C2 BioCarta pathway	BIOCARTA_GLEEVEC_PATHWAY	1.69	0.0072	***0.0417***
C5 GO Biological Process	GOBP_TRANSCRIPTION_INITIATION_FROM_RNA_POLYMERASE_II_PROMOTER	1.69	0.0000	***0.0417***

**Group**	**Gene Set Name**	**NES**	***p***	**FDR *q***
		**post, trained vs control**		

C2 PID pathway	PID_RHOA_REG_PATHWAY	1.69	0.0053	***0.0418***
C2 WikiPathways pathway	WP_PRION_DISEASE_PATHWAY	1.69	0.0130	***0.0420***
C2 WikiPathways pathway	WP_DNA_DAMAGE_RESPONSE_ONLY_ATM_DEPENDENT	1.69	0.0032	***0.0421***
C2 Reactome pathway	REACTOME_SCF_SKP2_MEDIATED_DEGRADATION_OF_P27_P21	1.69	0.0018	***0.0423***
C5 GO Biological Process	GOBP_I_KAPPAB_KINASE_NF_KAPPAB_SIGNALING	1.69	0.0000	***0.0424***
C2 Reactome pathway	REACTOME_TRANSLATION	1.69	0.0000	***0.0425***
C5 GO Biological Process	GOBP_ER_NUCLEUS_SIGNALING_PATHWAY	1.69	0.0018	***0.0426***
C2 Reactome pathway	REACTOME_APOPTOSIS	1.69	0.0000	***0.0427***
C5 GO Biological Process	GOBP_PEPTIDYL_LYSINE_METHYLATION	1.69	0.0000	***0.0427***
C5 GO Biological Process	GOBP_MAST_CELL_ACTIVATION	1.68	0.0017	***0.0428***
C2 Reactome pathway	REACTOME_REGULATION_OF_TP53_EXPRESSION_AND_DEGRADATION	1.69	0.0210	***0.0428***
C5 GO Biological Process	GOBP_ANTIGEN_PROCESSING_AND_PRESENTATION_OF_PEPTIDE_ANTIGEN_VIA_MHC_CLASS_I	1.69	0.0286	***0.0429***
C2 KEGG pathway	KEGG_CELL_CYCLE	1.68	0.0000	***0.0429***
C5 GO Biological Process	GOBP_RESPONSE_TO_PROSTAGLANDIN_E	1.69	0.0071	***0.0429***
Hallmark	HALLMARK_MYC_TARGETS_V2	1.69	0.0074	***0.0429***
C5 GO Biological Process	GOBP_PHOSPHOLIPID_CATABOLIC_PROCESS	1.69	0.0054	***0.0429***
C2 Reactome pathway	REACTOME_JNK_C_JUN_KINASES_PHOSPHORYLATION_AND_ACTIVATION_MEDIATED_BY_ACTIVATED_HUMAN_TAK1	1.69	0.0113	***0.0429***
C5 GO Biological Process	GOBP_NATURAL_KILLER_CELL_ACTIVATION	1.68	0.0017	***0.0429***
C2 Reactome pathway	REACTOME_CONSTITUTIVE_SIGNALING_BY_AKT1_E17K_IN_CANCER	1.69	0.0055	***0.0429***
C5 GO Molecular Function	GOMF_MHC_CLASS_I_PROTEIN_BINDING	1.69	0.0092	***0.0429***
C2 Reactome pathway	REACTOME_CYTOSOLIC_TRNA_AMINOACYLATION	1.69	0.0167	***0.0429***
C2 Reactome pathway	REACTOME_RESPIRATORY_ELECTRON_TRANSPORT	1.69	0.0017	***0.0430***
C5 GO Biological Process	GOBP_PROTEIN_STABILIZATION	1.69	0.0000	***0.0430***
C2 Reactome pathway	REACTOME_SIGNALING_BY_TGFB_FAMILY_MEMBERS	1.68	0.0034	***0.0431***
C5 GO Biological Process	GOBP_POSITIVE_REGULATION_OF_TELOMERE_MAINTENANCE	1.68	0.0073	***0.0431***
C5 GO Biological Process	GOBP_REGULATION_OF_LYMPHOCYTE_DIFFERENTIATION	1.68	0.0000	***0.0432***
C2 BioCarta pathway	BIOCARTA_IL2_PATHWAY	1.68	0.0145	***0.0432***
C5 GO Cellular Component	GOCC_PML_BODY	1.68	0.0000	***0.0432***
C2 Reactome pathway	REACTOME_RHO_GTPASE_EFFECTORS	1.68	0.0000	***0.0432***
C2 WikiPathways pathway	WP_FOLLICLE_STIMULATING_HORMONE_FSH_SIGNALING_PATHWAY	1.68	0.0071	***0.0432***
C5 GO Molecular Function	GOMF_GTPASE_ACTIVATOR_ACTIVITY	1.68	0.0000	***0.0432***

C2 WikiPathways pathway	WP_GLYCOGEN_SYNTHESIS_AND_DEGRADATION	1.68	0.0092	***0.0432***
C5 GO Cellular Component	GOCC_PROTEIN_ACETYLTRANSFERASE_COMPLEX	1.68	0.0000	***0.0433***
C5 GO Biological Process	GOBP_PHAGOCYTOSIS	1.68	0.0016	***0.0433***
C5 GO Biological Process	GOBP_INNATE_IMMUNE_RESPONSE_ACTIVATING_SIGNAL_TRANSDUCTION	1.68	0.0092	***0.0433***
C2 PID pathway	PID_CASPASE_PATHWAY	1.68	0.0036	***0.0433***
C2 Reactome pathway	REACTOME_NUCLEAR_ENVELOPE_NE_REASSEMBLY	1.68	0.0000	***0.0433***
C5 GO Cellular Component	GOCC_ENDOPEPTIDASE_COMPLEX	1.68	0.0037	***0.0434***
C5 GO Biological Process	GOBP_POSITIVE_REGULATION_OF_DNA_TEMPLATED_TRANSCRIPTION_INITIATION	1.68	0.0018	***0.0434***
C2 Reactome pathway	REACTOME_ZBP1_DAI_MEDIATED_INDUCTION_OF_TYPE_I_IFNS	1.68	0.0092	***0.0434***
C2 PID pathway	PID_TRAIL_PATHWAY	1.68	0.0132	***0.0434***
C5 GO Cellular Component	GOCC_NUCLEAR_SPECK	1.68	0.0000	***0.0435***

Group	Gene Set Name	NES	*p*	FDR *q*
		**post, trained vs control**		
C5 GO Biological Process	GOBP_B_CELL_PROLIFERATION	1.68	0.0018	***0.0435***
C2 Reactome pathway	REACTOME_PROTEIN_UBIQUITINATION	1.68	0.0018	***0.0435***
C5 GO Biological Process	GOBP_IMPORT_INTO_NUCLEUS	1.68	0.0017	***0.0436***
C2 KEGG pathway	KEGG_GRAFT_VERSUS_HOST_DISEASE	1.68	0.0147	***0.0436***
C5 GO Cellular Component	GOCC_SPECIFIC_GRANULE	1.68	0.0000	***0.0438***
C5 GO Biological Process	GOBP_RUFFLE_ORGANIZATION	1.68	0.0000	***0.0439***
C5 GO Biological Process	GOBP_POSITIVE_REGULATION_OF_UBIQUITIN_DEPENDENT_PROTEIN_CATABOLIC_PROCESS	1.68	0.0017	***0.0442***
C5 GO Cellular Component	GOCC_TERTIARY_GRANULE_LUMEN	1.68	0.0000	***0.0442***
C5 GO Biological Process	GOBP_NEGATIVE_REGULATION_OF_IMMUNE_SYSTEM_PROCESS	1.68	0.0000	***0.0442***
C2 Reactome pathway	REACTOME_TICAM1_RIP1_MEDIATED_IKK_COMPLEX_RECRUITMENT	1.68	0.0095	***0.0446***
C5 GO Biological Process	GOBP_PROTEIN_DESTABILIZATION	1.68	0.0075	***0.0446***
C5 GO Molecular Function	GOMF_ISOPRENOID_BINDING	-2.09	0.0000	***0.0446***
C2 Reactome pathway	REACTOME_INTERLEUKIN_10_SIGNALING	1.68	0.0137	***0.0446***
C2 Reactome pathway	REACTOME_REGULATION_OF_PTEN_GENE_TRANSCRIPTION	1.68	0.0018	***0.0446***
C2 KEGG pathway	KEGG_LEISHMANIA_INFECTION	1.67	0.0036	***0.0446***
C5 GO Biological Process	GOBP_RIBONUCLEOPROTEIN_COMPLEX_BIOGENESIS	1.68	0.0000	***0.0447***
C2 Reactome pathway	REACTOME_CROSS_PRESENTATION_OF_SOLUBLE_EXOGENOUS_ANTIGENS_ENDOSOMES	1.67	0.0053	***0.0447***

C5 GO Biological Process	GOBP_RESPIRATORY_BURST	1.67	0.0037	***0.0447***
C2 BioCarta pathway	BIOCARTA_PTEN_PATHWAY	1.67	0.0108	***0.0447***
C5 GO Cellular Component	GOCC_GOLGI_ASSOCIATED_VESICLE	1.67	0.0019	***0.0448***
C5 GO Molecular Function	GOMF_HSP70_PROTEIN_BINDING	1.67	0.0018	***0.0448***
C5 GO Cellular Component	GOCC_ENDOSOME_MEMBRANE	1.67	0.0000	***0.0448***
C5 GO Biological Process	GOBP_CELLULAR_RESPONSE_TO_MOLECULE_OF_BACTERIAL_ORIGIN	1.67	0.0000	***0.0448***
C5 GO Cellular Component	GOCC_IMMUNOLOGICAL_SYNAPSE	1.67	0.0038	***0.0448***
C5 GO Biological Process	GOBP_MYELOID_CELL_ACTIVATION_INVOLVED_IN_IMMUNE_RESPONSE	1.67	0.0000	***0.0449***
C5 GO Biological Process	GOBP_MONOVALENT_INORGANIC_ANION_HOMEOSTASIS	1.67	0.0221	***0.0451***
C2 WikiPathways pathway	WP_HEAD_AND_NECK_SQUAMOUS_CELL_CARCINOMA	1.67	0.0035	***0.0451***
C2 KEGG pathway	KEGG_N_GLYCAN_BIOSYNTHESIS	1.67	0.0112	***0.0453***
C2 Reactome pathway	REACTOME_OXIDATIVE_STRESS_INDUCED_SENESCENCE	1.67	0.0018	***0.0453***
C2 Reactome pathway	REACTOME_SIGNALING_BY_WNT	1.67	0.0000	***0.0453***
Hallmark	HALLMARK_IL2_STAT5_SIGNALING	1.67	0.0016	***0.0453***
C5 GO Biological Process	GOBP_REGULATION_OF_CARBOHYDRATE_CATABOLIC_PROCESS	1.67	0.0112	***0.0454***
C5 GO Biological Process	GOBP_COTRANSLATIONAL_PROTEIN_TARGETING_TO_MEMBRANE	1.67	0.0114	***0.0455***
C5 GO Biological Process	GOBP_REGULATION_OF_LEUKOCYTE_PROLIFERATION	1.67	0.0000	***0.0455***
C5 GO Cellular Component	GOCC_PRERIBOSOME	1.67	0.0035	***0.0455***
C5 GO Biological Process	GOBP_REGULATION_OF_PROTEASOMAL_UBIQUITIN_DEPENDENT_PROTEIN_CATABOLIC_PROCESS	1.67	0.0000	***0.0457***
C5 GO Biological Process	GOBP_REGULATION_OF_TRANSCRIPTION_OF_NUCLEOLAR_LARGE_RRNA_BY_RNA_POLYMERASE_I	1.67	0.0057	***0.0457***
C2 Reactome pathway	REACTOME_TRANSCRIPTIONAL_REGULATION_BY_SMALL_RNAS	1.67	0.0071	***0.0458***
C2 Reactome pathway	REACTOME_GOLGI_ASSOCIATED_VESICLE_BIOGENESIS	1.67	0.0071	***0.0458***
C2 WikiPathways pathway	WP_MICRORNAS_IN_CARDIOMYOCYTE_HYPERTROPHY	1.67	0.0000	***0.0458***
C5 GO Biological Process	GOBP_REGULATION_OF_OXIDATIVE_STRESS_INDUCED_INTRINSIC_APOPTOTIC_SIGNALING_PATHWAY	1.67	0.0055	***0.0459***
C5 GO Biological Process	GOBP_T_CELL_SELECTION	1.67	0.0035	***0.0460***

**Group**	**Gene Set Name**	**NES**	***p***	**FDR *q***
		**post, trained vs control**		
C5 GO Molecular Function	GOMF_TRANSLATION_REGULATOR_ACTIVITY	1.67	0.0000	***0.0460***
C5 GO Biological Process	GOBP_MONONUCLEAR_CELL_DIFFERENTIATION	1.67	0.0000	***0.0460***

C5 GO Biological Process	GOBP_MRNA_SPLICE_SITE_SELECTION	1.67	0.0107	***0.0461***
C5 GO Biological Process	GOBP_INTRINSIC_APOPTOTIC_SIGNALING_PATHWAY_IN_RESPONSE_TO_DNA_DAMAGE	1.67	0.0017	***0.0461***
C5 GO Biological Process	GOBP_NCRNA_PROCESSING	1.67	0.0000	***0.0461***
C5 GO Biological Process	GOBP_POSITIVE_REGULATION_OF_CELL_ADHESION_MEDIATED_BY_INTEGRIN	1.67	0.0096	***0.0462***
C5 GO Cellular Component	GOCC_INTRINSIC_COMPONENT_OF_ORGANELLE_MEMBRANE	1.67	0.0000	***0.0462***
C2 Reactome pathway	REACTOME_TRAF6_MEDIATED_NF_KB_ACTIVATION	1.67	0.0147	***0.0463***
C5 GO Biological Process	GOBP_RIBOSE_PHOSPHATE_BIOSYNTHETIC_PROCESS	1.66	0.0017	***0.0465***
C5 GO Molecular Function	GOMF_NUCLEOCYTOPLASMIC_CARRIER_ACTIVITY	1.66	0.0055	***0.0467***
C2 Reactome pathway	REACTOME_CYCLIN_A_B1_B2_ASSOCIATED_EVENTS_DURING_G2_M_TRANSITION	1.66	0.0112	***0.0467***
C5 GO Biological Process	GOBP_POSITIVE_REGULATION_OF_ALPHA_BETA_T_CELL_DIFFERENTIATION	1.66	0.0054	***0.0468***
C2 PID pathway	PID_CDC42_PATHWAY	1.66	0.0053	***0.0468***
C5 GO Biological Process	GOBP_FATTY_ACYL_COA_METABOLIC_PROCESS	1.66	0.0200	***0.0468***
C5 GO Biological Process	GOBP_NEGATIVE_REGULATION_OF_MAP_KINASE_ACTIVITY	1.66	0.0087	***0.0468***
C2 WikiPathways pathway	WP_FBXL10_ENHANCEMENT_OF_MAPERK_SIGNALING_IN_DIFFUSE_LARGE_BCELL_LYMPHOMA	1.66	0.0184	***0.0468***
C5 GO Cellular Component	GOCC_CATALYTIC_STEP_2_SPLICEOSOME	1.66	0.0017	***0.0469***
C2 WikiPathways pathway	WP_CHEMOKINE_SIGNALING_PATHWAY	1.66	0.0000	***0.0469***
C5 GO Molecular Function	GOMF_PHOSPHATIDYLINOSITOL_KINASE_ACTIVITY	1.66	0.0153	***0.0470***
C5 GO Cellular Component	GOCC_GOLGI_APPARATUS_SUBCOMPARTMENT	1.66	0.0000	***0.0474***
C5 GO Biological Process	GOBP_POSITIVE_REGULATION_OF_CELLULAR_AMIDE_METABOLIC_PROCESS	1.66	0.0000	***0.0474***
C5 GO Molecular Function	GOMF_GENERAL_TRANSCRIPTION_INITIATION_FACTOR_BINDING	1.66	0.0089	***0.0474***
C5 GO Biological Process	GOBP_PROTEIN_LOCALIZATION_TO_PHAGOPHORE_ASSEMBLY_SITE	1.66	0.0199	***0.0475***
C5 GO Cellular Component	GOCC_U12_TYPE_SPLICEOSOMAL_COMPLEX	1.66	0.0143	***0.0476***
C5 GO Molecular Function	GOMF_PROTEIN_TRANSPORTER_ACTIVITY	1.66	0.0107	***0.0476***
C5 GO Biological Process	GOBP_NEGATIVE_REGULATION_OF_RESPONSE_TO_DNA_DAMAGE_STIMULUS	1.66	0.0000	***0.0476***
C5 GO Molecular Function	GOMF_CYSTEINE_TYPE_ENDOPEPTIDASE_REGULATOR_ACTIVITY_INVOLVED_IN_APOPTOTIC_PROCESS	1.66	0.0091	***0.0476***
C5 GO Biological Process	GOBP_MATURATION_OF_SSU_RRNA	1.66	0.0095	***0.0476***
C2 BioCarta pathway	BIOCARTA_EIF4_PATHWAY	1.66	0.0108	***0.0476***
C2 WikiPathways pathway	WP_AGERAGE_PATHWAY	1.66	0.0017	***0.0477***

C5 GO Cellular Component	GOCC_GOLGI_ASSOCIATED_VESICLE_MEMBRANE	1.66	0.0035	***0.0477***
C2 KEGG pathway	KEGG_RNA_DEGRADATION	1.66	0.0053	***0.0477***
C5 GO Biological Process	GOBP_ANTIGEN_PROCESSING_AND_PRESENTATION_OF_EXOGENOUS_ANTIGEN	1.66	0.0074	***0.0478***
C2 PID pathway	PID_INSULIN_PATHWAY	1.66	0.0108	***0.0478***
C2 WikiPathways pathway	WP_NOTCH_SIGNALING	1.66	0.0107	***0.0478***
C5 GO Biological Process	GOBP_NEGATIVE_REGULATION_OF_RELEASE_OF_CYTOCHROME_C_FROM_MITOCHONDRIA	1.66	0.0219	***0.0482***
C2 PID pathway	PID_P38_MK2_PATHWAY	1.66	0.0134	***0.0482***
C5 GO Biological Process	GOBP_NEGATIVE_REGULATION_OF_PROTEIN_ACETYLATION	1.66	0.0150	***0.0482***
C2 Reactome pathway	REACTOME_E3_UBIQUITIN_LIGASES_UBIQUITINATE_TARGET_PROTEINS	1.66	0.0093	***0.0486***
C5 GO Molecular Function	GOMF_PHOSPHOPROTEIN_BINDING	1.66	0.0018	***0.0486***
C5 GO Biological Process	GOBP_ESTABLISHMENT_OF_PROTEIN_LOCALIZATION_TO_CHROMOSOME	1.66	0.0146	***0.0487***
C5 GO Cellular Component	GOCC_MICROBODY	1.65	0.0000	***0.0489***

**Group**	**Gene Set Name**	**NES**	***p***	**FDR *q***
C5 GO Biological Process	GOBP_REGULATION_OF_IMMUNE_EFFECTOR_PROCESS	1.65	0.0000	***0.0490***
C2 WikiPathways pathway	WP_NUCLEOTIDE_EXCISION_REPAIR	1.65	0.0127	***0.0491***
C2 Reactome pathway	REACTOME_DNA_REPAIR	1.65	0.0000	***0.0491***
C5 GO Biological Process	GOBP_REGULATION_OF_DNA_REPAIR	1.65	0.0000	***0.0492***
C2 Reactome pathway	REACTOME_INTERLEUKIN_12_SIGNALING	1.65	0.0000	***0.0492***
C5 GO Biological Process	GOBP_LEUKOCYTE_HOMEOSTASIS	1.65	0.0017	***0.0495***
C2 Reactome pathway	REACTOME_BBSOME_MEDIATED_CARGO_TARGETING_TO_CILIUM	1.65	0.0127	***0.0497***
C5 GO Biological Process	GOBP_NEGATIVE_REGULATION_OF_REACTIVE_OXYGEN_SPECIES_METABOLIC_PROCESS	1.65	0.0108	***0.0498***
C2 KEGG pathway	KEGG_RENAL_CELL_CARCINOMA	1.65	0.0052	***0.0498***
C5 GO Biological Process	GOBP_HISTONE_METHYLATION	1.65	0.0000	***0.0498***
C5 GO Biological Process	GOBP_REGULATION_OF_GENERATION_OF_PRECURSOR_METABOLITES_AND_ENERGY	1.65	0.0000	***0.0498***
C5 GO Cellular Component	GOCC_TERTIARY_GRANULE	1.65	0.0000	***0.0498***
C2 BioCarta pathway	BIOCARTA_CTCF_PATHWAY	1.65	0.0209	***0.0499***
C2 WikiPathways pathway	WP_FACTORS_AND_PATHWAYS_AFFECTING_INSULINLIKE_GROWTH_FACTOR_IGF1AKT_SIGNALING	1.65	0.0129	***0.0499***
C5 GO Biological Process	GOBP_REGULATION_OF_TRANSCRIPTION_BY_RNA_POLYMERASE_I	1.65	0.0035	***0.0500***
C5 GO Biological Process	GOBP_COENZYME_A_METABOLIC_PROCESS	1.65	0.0168	***0.0500***
